# A Tetra-Orthogonal Strategy for the Efficient Synthesis of Scaffolds Based on Cyclic Peptides

**DOI:** 10.1007/s10989-017-9642-0

**Published:** 2017-11-01

**Authors:** Nitin Jain, Simon H. Friedman

**Affiliations:** Division of Pharmaceutical Sciences, School of Pharmacy, University of Missouri-Kansas City, 2464 Charlotte Street, Kansas City, MO 64108 USA

**Keywords:** Cyclic peptide, Parallel library, Scaffold, Quinoxaline, Orthogonal, On-resin cyclization

## Abstract

We have developed a straightforward and robust strategy for synthesizing a family of cyclic peptide scaffolds for the presentation of defined moieties in a wide range of orientations. Specifically we are exploring quinoxaline as the moiety, as a potential nucleic acid binding motif. The method requires the use of four degrees of orthogonality, which in turn allow the extension of the main chain, incorporation of the target side chains, on-resin cyclization, and the revelation of an amino group upon cleavage to increase solubility. We show that related approaches fail for a range of reasons, including the failure of cyclization. Following the optimization of the approach with a single cyclic peptide, we synthesized a family of all possible bis and tris quinoxaline adducts showing by ESI–MS that the desired full length cyclic product is produced in a majority of cases.

**Electronic supplementary material:**

The online version of this article (10.1007/s10989-017-9642-0) contains supplementary material, which is available to authorized users.

## Introduction

There is a need for conveniently synthesized scaffolds that can present multiple groups and allow for the variation of the groups’ geometry relative to each other.(Baldini et al. 2007; Mammen et al. 1998; Wu et al. 2005) Multivalent presentation is important in diverse fields such as immunology and cell recognition, and both affinity and specificity are strongly linked to the simultaneous binding of weak ligands (Badjiƒá et al. 2005; Kiessling et al. 2000, 2006; Lundquist and Toone 2002; Mammen et al. 1998; Strong and Kiessling 1999). This simultaneous binding in turn is linked to the spatial relationships that exist between the ligands. In this vein, we sought a convenient scaffold upon which we could vary the presentation of weakly intercalating groups, to recognize nucleic acids. Cyclic peptides are ideal for this purpose, as they can be made with high yielding reactions and have very high potential structural and conformational diversity, while having limited conformational entropy costs associated with binding (Baeriswyl and Heinis 2013; Bogdanowich-Knipp et al. 1999; Cardote and Ciulli 2016; Craik et al. 2002; Deber et al. 1976; Driggers et al. 2008; Joo 2012; Lambert et al. 2001; Schlippe et al. 2012; Tsomaia 2015; Zorzi et al. 2017).

The main challenge for the use of cyclic peptides is to establish synthetic approaches that are high yielding and that allow for the installation of the presentation groups at any position, prior to cyclization. In this work we describe the successful identification of such an approach. It utilizes four degrees of orthogonality to allow the incorporation of the presentation group (in our case quinoxaline carboxylic acid), into an amine position, followed by cyclization, and uncovering of a terminal amine, to increase solubility. We optimized the approach using a defined hexa-peptide cycle with two presented quinoxalines, and then used the approach to make a screening library of 20 cyclic heptapeptides representing all possible two and three group presenting scaffolds. Almost all showed a majority cyclic versus linear form, and were synthesized with good crude purity.

## Results and Discussion

**Scheme 1:**
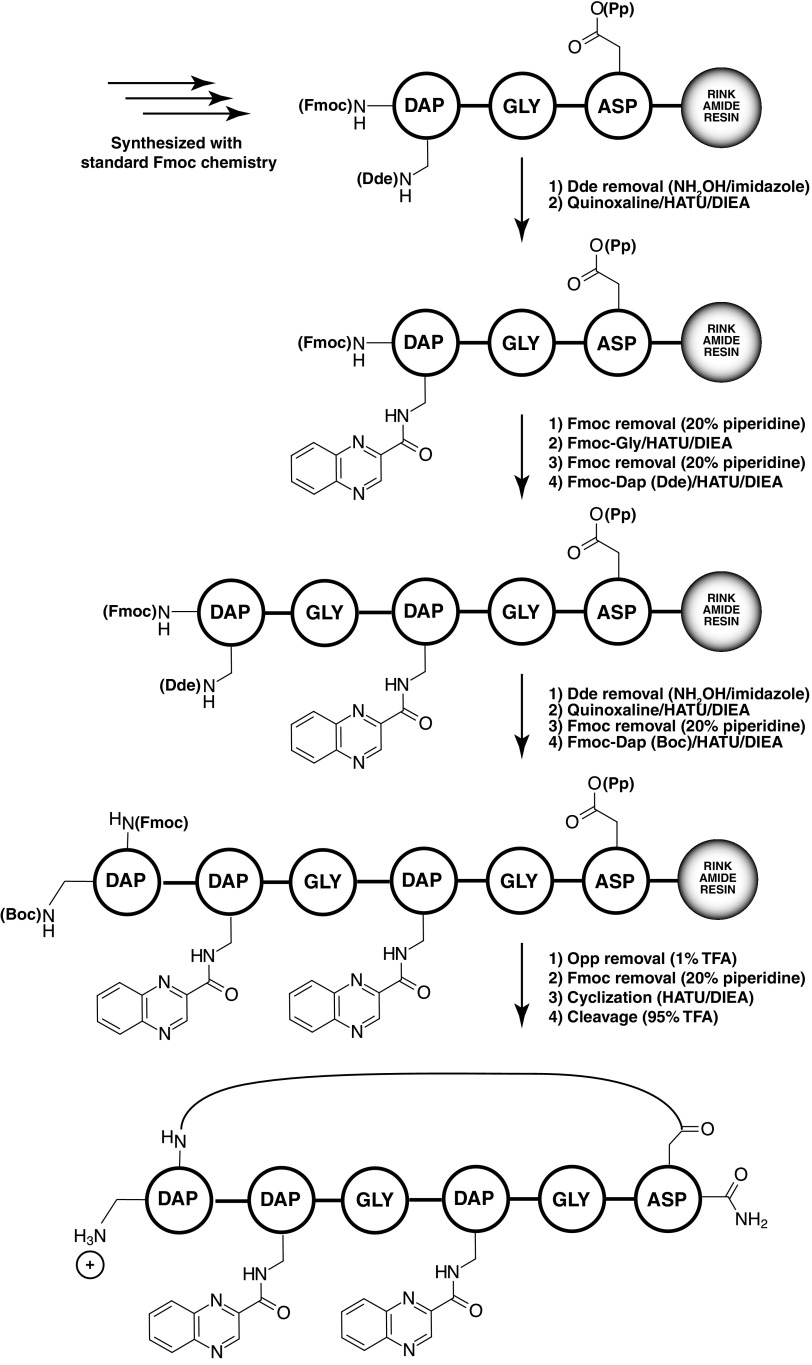
Successful scaffold synthesis approach

The overall aim of our work was to create a robust scaffold that would allow for the variation in the geometry of presentation of multiple moieties, specifically quinoxaline 2 carboxylic acid. The quinoxaline moiety is a planar aromatic system that is found in the nucleic acid binding natural product triostin. We wanted to create families of molecules that presented quinoxaline in a range of geometries. Because of this, we were not wedded to a specific motif or synthetic strategy, as long as the final molecules allowed for easy structural variation and sufficient purity/yield for screening. We found that only a narrow set of conditions allowed for a successful and robust synthesis. These conditions are shown in Scheme 1. To identify the successful conditions, we examined multiple methods to synthesize a specific sequence containing six amino acids, and two sites of quinoxaline incorporation. We will briefly describe some of the approaches that were not successful, followed by the successful method.

**Scheme 2:**
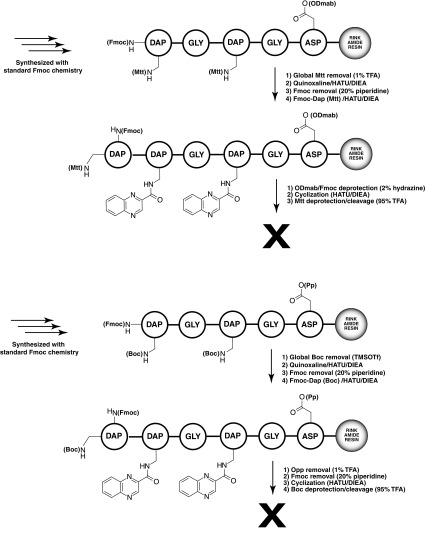
Unsuccessful scaffold synthesis approaches

In all methods we examined, we attempted to create the final cycle through the side chain of an aspartic acid in the first synthetic position, and the alpha terminal amine of the final amino acid in the chain, diamino propionic acid (Dap). In the first approach we attempted (Scheme 2, top), we used Dap groups with their beta amino functionality protected by the Mtt group. After the entire linear chain was synthesized, we globally deprotected these Mtt groups (using 1% TFA), and the resulting exposed amino groups were acylated with quinoxaline 2 carboxylic acid. The ODmab group protecting the aspartyl carboxyl and the terminal Fmoc amino groups were then simultaneously removed using 2% hydrazine, followed by HATU driven cyclization between this carboxyl and amino group. Analysis of this reaction product showed no full length product, linear or cyclic. Masses consistent with incomplete ODmab deprotection, as well as aspartimide formation and capped linear form were observed (data not shown).

A second unsuccessful approach is shown in Scheme 2, bottom. In this approach, the Dap side chain amino groups were protected by Boc groups, and the aspartic acid side chain was protected by the phenyl isopropyl (Opp) group. Five amino acids were incorporated, leaving out the final Dap. The Boc groups were selectively deprotected using trimethyl silyl triflate, after which quinoxaline 2 carboxylic acid was incorporated into the exposed amino groups. The final Dap was introduced into the terminal, and the Fmoc protected alpha amine was deprotected with 20% piperidine. The Opp group on aspartic acid was then deprotected using 1% TFA, and the cyclization effected with HATU. Analysis of the cleaved and washed final product by LC–MS showed no identifiable linear or cyclic product. It is possible that this strategy failed because of incomplete orthogonality in the deprotection of Boc in the presence of the Opp group. We were inspired in part by the work of Cavelier who showed that TMSOTf can remove Boc groups without completely cleaving peptides from Wang resin.(Lejeune et al. 2003).

The final successful strategy (Scheme 1), incorporated elements from these unsuccessful methods while introducing a fourth degree of orthogonality, which ultimately allowed for high yields of full cyclized product. In this approach, the amino acids were installed using standard Fmoc chemistry coupled with HATU. All Dap amino acids (intended as the base upon which to incorporate the quinoxaline 2 carboxylic acid) used Dde side chain protection. As each mid-chain Dap was incorporated, the Dde group was removed by reaction with the method of Bradley and coworkers, namely hydroxylamine/imidazole.(Diaz-Mochon et al. 2004; Wilhelm et al. 2000). This exposed the beta amino group, which was then immediately acylated with quinoxaline 2 carboxylic acid. We considered using a global Dde deprotection at the end of synthesis but were concerned with the possibility of Dde migration.(Wilhelm et al. 2000).

The sixth and final amino acid incorporated was Dap with a standard alpha amino Fmoc protection but with a side chain protected by a Boc group. If we had used a Dde group instead for the side chain amine protection, we again would have run the risk of Dde migration. As before, we deprotected the Opp protected aspartic acid side chain with 1% TFA, the terminal alpha amino group with 20% piperidine, and performed cyclization using HATU. The final product was cleaved from resin using 90% TFA/H_2_O. This also cleaved the Boc group from the side chain amine of the terminal Dap group. This amino group was included to introduce positive charge, needed for solubility and to potentially enhance binding to nucleic acid targets.

**Fig. 1:**
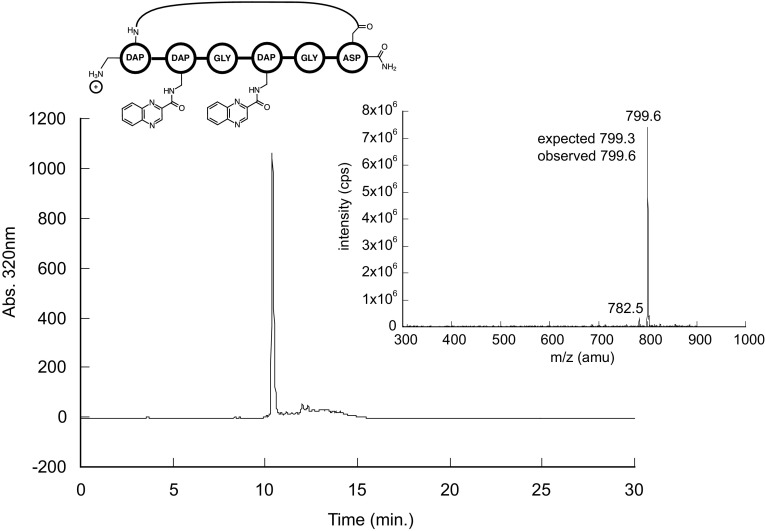
Characterization of cyclic hexapeptide synthesized with successful scaffold synthesis approach. HPLC trace at 320 nm (bottom) and direct infusion ESI–MS (inset) of peptide

Using this strategy, we observed the desired cyclized full length product. Figure 1 shows both the HPLC trace and the ESI mass spectrum. The 320 nm chromatogram (lambda max of quinoxaline) shows a single sharp peak, and the ESI mass spectrum for the product indicates an MH+m/z of 799.6, with the expected MH+m/z being 799.3.

**Fig. 2:**
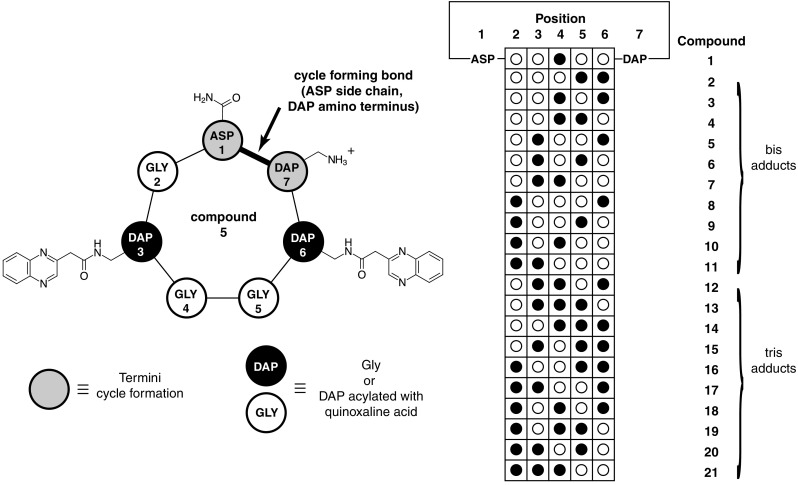
Setup of heptapeptide library

With these successful conditions in place, we then focused on creating a library of compounds, in which the moieties being presented (in our case, quinoxaline) were systematically varied in their position in the motif. To increase diversity and to examine the robustness of the approach with a related motif, we expanded the peptide cycle to seven amino acids, a larger and potentially more challenging cycle to form. As before, the first amino acid (aspartic acid) side chain carboxyl, and the last amino acid (Dap) terminal amine were used to effect the final cyclization. Between them, we placed five positions that were either presentation positions (Dap acylated with quinoxaline) or spacer positions (Gly). With this motif we had theoretically ten possible bis products (5 × 4/2!) and ten possible tris products (5 × 4 × 3/3!), all of which we synthesized. In addition, we synthesized one product with a single Dap in the motif. These structures are summarized in Fig. 2.

**Fig. 3:**
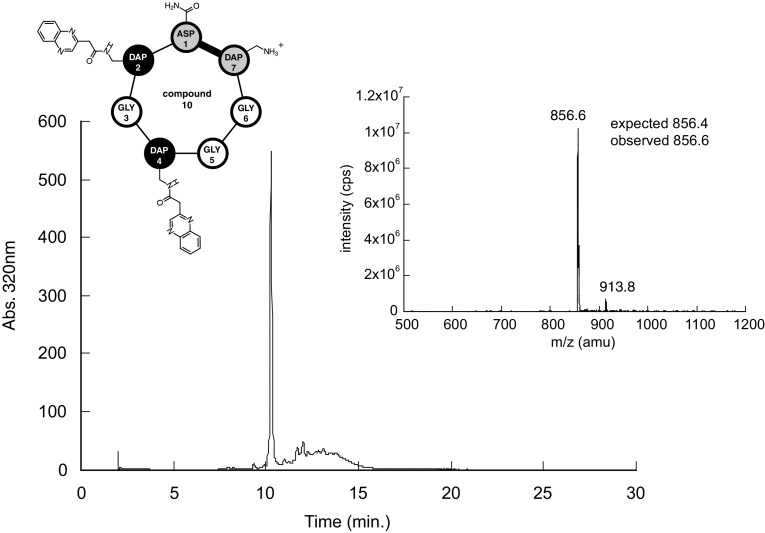
Characterization of representative cyclic peptide (compound 10) from heptapeptide library. HPLC trace at 320 nm (bottom) and ESI–MS (inset) of peptide. A 210 nm HPLC trace may be found in supporting information

A representative result of the synthesis is shown in Fig. 3 (compound 10). Both HPLC and MS of the crude reaction products are shown. The HPLC chromatogram shows a major sharp peak, and the ESI–MS spectrum confirms the expected mass of the cyclized product (MH+expected 856.4, observed 856.6). For these studies we analyzed the crude reaction product, to realistically indicate the side products present. The complete HPLC and ESI–MS data for the remaining 19 peptides are shown in the Supporting Information, and summarized in Table 1. The synthetic approach proved to be robust, and all expected species were confirmed to be synthesized by MS. In a large majority of the cases, the major species observed in MS is the cyclized form, with small amounts showing the expected+18 mass of the linear form. In four of the tris-adducts, we observe both cyclic and linear forms present, with linear being in the majority. This is reasonable given the greater steric crowding found in tris adducts, making cyclization more challenging. In approximately half of the bis-quinoxaline species we observe a single peak in the HPLC chromatogram. In the other half of cases with bis-quinoxalines and a majority of tris-quinoxalines we observe a doubling of the HPLC peak. This may be due to the linear/cyclic issue just described.

**Table 1:** Summary of mass spectrometric analytical data for all members of the bis and tris quinoxaline library

Species #	Sequence (middle Dap positions ultimately modified with quinoxaline acid)	# Of mods	Largest MS signal (amu)	Expected MS signal (amu)	Yield (%)^a^	% HPLC purity (320 nm)
1	Asp-Gly-Gly-Dap-Gly-Gly-Dap	1	671.5	671.4	41	84
2	Asp-Gly-Gly-Gly-Dap-Dap-Dap	2	856.5	856.5	39	35
3	Asp-Gly-Gly-Dap-Gly-Dap-Dap	2	856.6	856.6	28	39
4	Asp-Gly-Gly-Dap-Dap-Gly-Dap	2	856.5	856.5	35	69
5	Asp-Gly-Dap-Gly-Gly-Dap-Dap	2	856.4	856.7	33	53
6	Asp-Gly-Dap-Gly-Dap-Gly-Dap	2	856.5	856.3	26	79
7	Asp-Gly-Dap-Dap-Gly-Gly-Dap	2	856.4	856.7	34	60
8	Asp-Dap-Gly-Gly-Gly-Dap-Dap	2	856.5	856.6	31	64
9	Asp-Dap-Gly-Gly-Dap-Gly-Dap	2	856.5	856.4	28	56
10	Asp-Dap-Gly-Dap-Gly-Gly-Dap	2	856.4	856.4	28	76
11	Asp-Dap-Dap-Gly-Gly-Gly-Dap	2	856.4	856.5	25	75
12	Asp-Gly-Dap-Dap-Gly-Dap-Dap	3	1041.5	1041.4	31	81
13	Asp-Gly-Dap-Dap-Dap-Gly-Dap	3	1041.6	1041.5	36	79
14	Asp-Gly-Gly-Dap-Dap-Dap-Dap	3	1041.5	1041.4	36	25
15	Asp-Gly-Dap-Gly-Dap-Dap-Dap	3	1041.6	1041.4	29	49
16	Asp-Dap-Gly-Gly-Dap-Dap-Dap	3	1041.5	1041.5	28	48
17	Asp-Dap-Dap-Gly-Gly-Dap-Dap	3	1059.5	1041.5	38	30
18	Asp-Dap-Gly-Dap-Gly-Dap-Dap	3	1041.5	1041.4	27	24
19	Asp-Dap-Gly-Dap-Dap-Gly-Dap	3	1059.4	1041.4	39	34
20	Asp-Dap-Dap-Gly-Dap-Gly-Dap	3	1059.4	1041.4	40	39
21	Asp-Dap-Dap-Dap-Gly-Gly-Dap	3	1059.4	1041.4	41	48

^a^Yield and purity determined as described in supporting information

## Conclusions

In this work we have identified a synthetic approach that allows for the synthesis of a set of cyclic peptide scaffolds for the presentation of specific groups in a complete and exhaustive manner. While there have been many methods described for synthesizing cyclic peptides (Bock et al. 2013; George et al. 2008; Jeon et al. 2009; White and Yudin 2011, 2012), atypically our approach allows for the convenient installation and variation of presented moieties during the synthesis. Although we focused on a specific group, the quinoxaline ring, the exact identity of this group can be varied with each presentation position, and can be essentially any carboxylic acid. This greatly expands the variability available. In addition, the ability to incorporate two and even three presented groups while still permitting efficient cyclization broadens the method’s utility.

The synthesis relies on four degree of orthogonality, allowing the building of the peptide main chain, installation of the target side chains, on-resin cyclization and finally revelation of an amine to increase solubility. We have made the complete and exhaustive set of 10 bis and 10 tris derivatives. The diversity of the motif can be expanded by using both L and D amino acids in the Dap and Asp positions, increasing diversity by a factor of 16 for the bis family and 32 for the tris family. Further diversity can be introduced through variation of the glycine positions to include the complete standard set of L and D amino acids. The final potential diversity of this cyclic family exceeds 20 million. We anticipate that the amino acid incorporation will not only introduce functional variation, but also significant conformational variation, as the effect of the amino acid side chains propagate through the overall structure. This relatively straightforward synthetic strategy represents a useful starting point for the creation of conformationally restricted molecules that can present desired moieties in a wide and diverse set of orientations. This, in turn, can make them useful for exploring biological systems in which such multivalent presentation is critical.

## Materials and Methods

Detailed synthetic procedures, and complete HPLC and MS data for all library members can be found in supporting information.

## Electronic supplementary material

Below is the link to the electronic supplementary material.

Supplementary material 1 (PDF 18450 KB)
