# Supplementary material for: A Tetra-Orthogonal Strategy for the Efficient Synthesis of Scaffolds Based on Cyclic Peptides
Source: Int J Pept Res Ther. 2017 Nov 1;24(4):535–42. doi: 10.1007/s10989-017-9642-0 (PMC6208650; doi:10.1007/s10989-017-9642-0)
Supplement: Supplementary file 1 — Supplementary material 1 (PDF 18450 KB) [file 10989_2017_9642_MOESM1_ESM.pdf]

Supporting Information

A Tetra-Orthogonal Strategy for the Efficient Synthesis of Scaffolds Based on Cyclic Peptides

Nitin Jain and Simon H. Friedman*

*e-mail: FriedmanS@umkc.edu

Contents

Materials and Methods **2**

Materials 2

Analytical Characterization 6

Analytical results **7**

HPLC chromatograms 7

ESI-MS analysis 15

References **25**

Materials and Methods

Materials

Fmoc-Asp(Opp)-OH and Fmoc-Dap(Dde)-OH were purchased from Chem-Impex International Inc. (Wood Dale, IL). Fmoc-Dap(Boc)-OH and NMP (N-methyl-2-pyrrolidone) were obtained from Advanced ChemTech (Louisville, KY, USA). HATU (O- (7-azabenzotriazole-1-yl)-N, N, N' N'-tetramethyluronium hexafluorophosphate) was purchased from Novabiochem. DIEA (N, N-diisopropylethylamine) and piperidine (99%) were obtained from Sigma Aldrich (St. Louis, MO, USA). Rink amide MBHA resin was obtained from EMD Biosciences (San Diego, CA, USA). TFA (99%) was purchased from Acros Organics (New Jersey, USA).

Method

Overview of approach: The synthesis uses four degrees of orthogonality using the protecting groups Opp, Dde, Fmoc and Boc. The Opp group was deprotected using 1% TFA in DCM. The Dde group was selectively deprotected in the presence of Fmoc using NH_2OH-Imidazole solution. The Fmoc group was selectively deprotected using 20% piperidine solution and finally the Boc group was deprotected using 95% TFA during the final cleavage. The final molecules had a net +1 charge.

Synthesis was performed on 15 mg of rink amide MBHA (0.7 mmole/gm.) resin in a 1.5 mL Eppendorf® microcentrifuge tube. Resin was first washed with NMP (300µL x 5 x 5 min). Fmoc deprotection was then performed using 300µL of 20% piperidine in two rounds of 7 min and 10 min respectively. Post deprotection, thorough washings (300µL NMP x 5 x 5 min) were done to remove excess piperidine. The free amine group

on the resin was then coupled with Fmoc-Asp(Opp)-OH. A concentration of 300 mM and a volume of 300 μL were used for the coupling step. HATU (300 mM) and DIEA (600 mM) were used as coupling reagents. The carboxylic acid of Fmoc-Asp(Opp)-OH was preactivated for 10 min following which acylation was done for 3 hours. After 3 hours, the reaction mixture was aspirated and the resin was washed with NMP (300μL x 5 x 5 min). Any unreacted amine on the resin was capped using freshly prepared capping solution containing 10% acetic anhydride and 5% DIEA in NMP for 15 min. NMP washes (300μL x 5 x 5 min) were done to remove any excess capping solution. The N^{α}-Fmoc group was then deprotected using 20% piperidine followed by NMP washes as mentioned above. At this stage the side chain carboxylic acid group was still protected with the phenylisopropyl ester group. Depending on the desired sequence, either Fmoc-Gly-OH or Fmoc-Dap(Dde)-OH was then coupled using the conditions described above for Fmoc-Asp(Opp)-OH. Following Dde couplings, the side chain protecting Dde group was selectively deprotected and acylated with quinoxaline-2-carboxylic. This was intentionally done to prevent the migration of the Dde group to α- position as has been reported in the literature. The Dde group was selectively deprotected using conditions that were developed by Bradley et al. for the selective deprotection of the Dde group in the presence of Fmoc group [1]. For this a stock solution of NH_2OH (1250mg) and imidazole (918mg) was prepared in 5 mL of NMP. The solution was sonicated to help dissolve the chemicals in the highly viscous solution. Once a clear solution was obtained, 5 volumes of this stock solution was mixed with 1 volume of methylene chloride. For the deprotection step, 250 μL of NH_2OH-Imidazole stock solution was mixed with 50 μL of methylene chloride and thoroughly mixed. The resulting 300 μL solution was then added

to the resin and the deprotection of Dde group was carried out for 3 hours. Post deprotection, NMP washes (300 μL x 10 x 5 min) were done to remove traces of NH_2OH-Imidazole solution. A total of 10 washes were done to accomplish this. Once the side chain amine was deprotected, it was then acylated with quinoxaline-2-carboxylic acid. The coupling solution (300μl) comprised quinoxaline-2-carboxylic acid (300mM), HATU (300 mM) and DIEA (600 mM). Preactivation was performed for 10 minutes followed by overnight (~12-15 hours) acylation. Post coupling, standard NMP washes were performed followed by capping and NMP washes. The Fmoc group was then selectively deprotected using standard 20% piperidine conditions. Additional rounds as needed of Fmoc-Gly-OH or Fmoc-Dap(Dde)-OH were then repeated using conditions mentioned above. The final residue incorporated was Fmoc-Dap(Boc)-OH and was used to achieve side chain to backbone cyclization. The N^{α} amine group of this terminal Dap was used for the side chain- backbone cyclization while the side chain amine group was used to help provide a net +1 charge to the molecule.

To achieve the on-resin cyclization, the Fmoc-Dap(Boc)-OH terminal α-NH_2 was first selectively deprotected using 300 μL of 20% piperidine in two rounds of 7 min and 10 min respectively. NMP (300 μL x 5 x 5 min) washes were done to remove any piperidine residue, followed by 3 DCM washes. The DCM washes were done to remove NMP and prepare the resin for the aspartic acid side chain deprotection. Finally, the phenylisopropyl ester group on the aspartic acid side chain carboxylic acid group was selectively deprotected using 1% TFA in DCM (300 μL x 13 x 2 min). Multiple DCM washes were done to remove traces of TFA followed by NMP washes (300μL x 5 x 5 min).

Once the aspartic acid side chain carboxylic acid and terminal Dap α-amine were deprotected, on-resin side chain to backbone cyclization was achieved using HATU and DIEA as coupling agents. 57mM peptide, 59.5mM HATU, 119mM DIEA. Unlike, normal couplings where the ratio of amino acid: HATU: DIEA were 1:1:2, a ratio of 1:1.05:2.1 was used for the cyclization step. Required amounts of HATU and DIEA were taken in 200 μL NMP. The carboxylic acid was activated in situ and cyclization reaction was carried out overnight. After overnight cyclization, resin was thoroughly washed with NMP and then with DCM.

After DCM washes, the resin was air dried and the test molecule was finally cleaved from the resin using 300 μL of cleavage cocktail that consisted of TFA: H_2O: TIS (95: 2.5: 2.5 volume ratio) for 3 hours. Post cleavage, the TFA solution was transferred to a microcentrifuge. TFA was removed using a stream of nitrogen followed by three rounds of ether trituration using ice cold ether to remove any traces of TFA. The resulting residue was dissolved in 50 μL DMSO for analytical characterization.

Analytical Characterization

Spectroscopy

UV-Vis analysis of the molecules was performed using a USB-2000 fiber optic spectrometer (Ocean Optics, Inc.) with a DT-Mini-B lamp source. The final yield of the molecules was calculated using a molar extinction coefficients of 7722 M^{-1} cm^{-1}; 15,444 M^{-1} cm^{-1} and 23,166 M^{-1} cm^{-1} for the mono, bis and tris molecules respectively.

HPLC analysis

HPLC analysis was performed on crude reaction products using a Hewlett Packard 1090 instrument with diode array detection. A Microsorb C8 (5 μm, 150 × 2.0 mm, Varian) column was used. A gradient from 0-100% solvent B (solvent A: 0.1% TFA/water, solvent B: acetonitrile) with a flow rate of 0.3ml/min over 30 min was used. Electrospray MS of the collective crude was performed in the positive ion mode using an ABI Q-Trap 2000 mass spectrometer. Reaction yields were assessed by determining the moles of quinoxaline present in the product and comparing these with the expected moles based on resin derivatization. Purity was estimated by comparing the HPLC integration of the tallest peak or peak cluster with the integration for the total chromatogram.

Analytical results

HPLC Chromatograms
210nm Chromatogram of Molecule 10

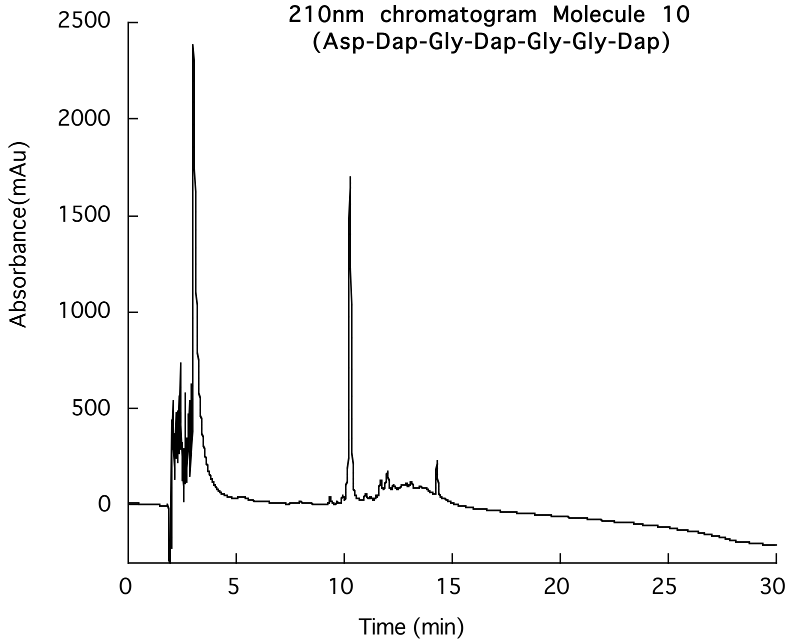

HPLC chromatograms: 21 crude products obtained at 320nm after TFA cleavage of the cyclic heptapeptide library.

Molecule 1: Asp-Gly-Gly-Dap-Gly-Gly-Dap

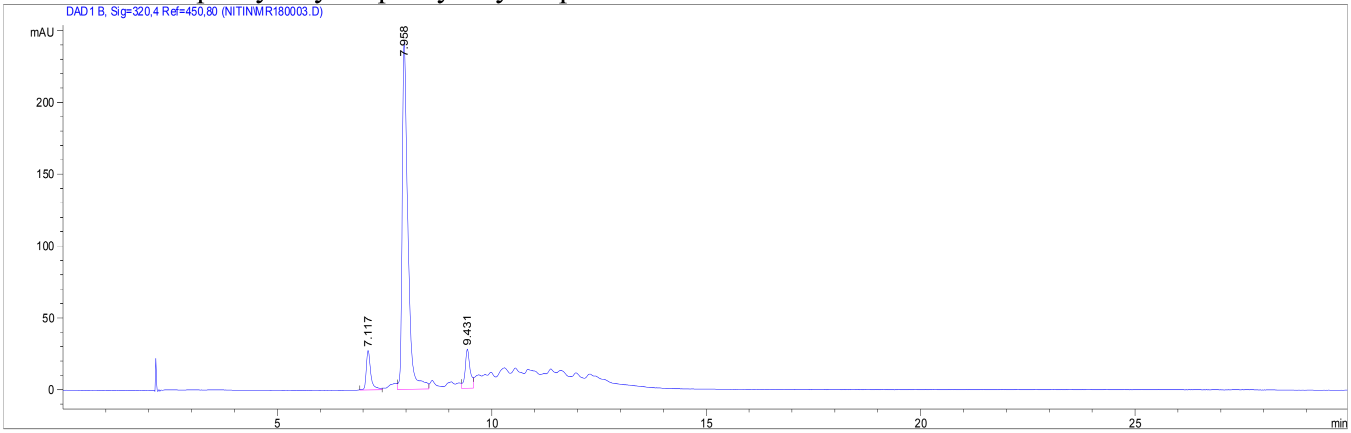

Molecule 2: Asp-Gly-Gly-Gly-Dap-Dap-Dap

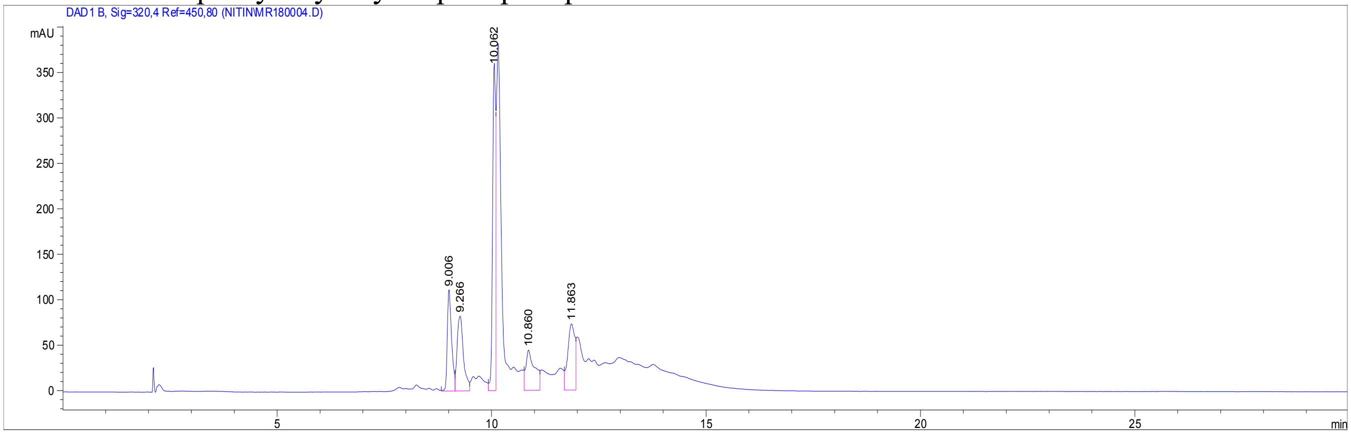

Molecule 3: Asp-Gly-Gly-Dap-Gly-Dap-Dap

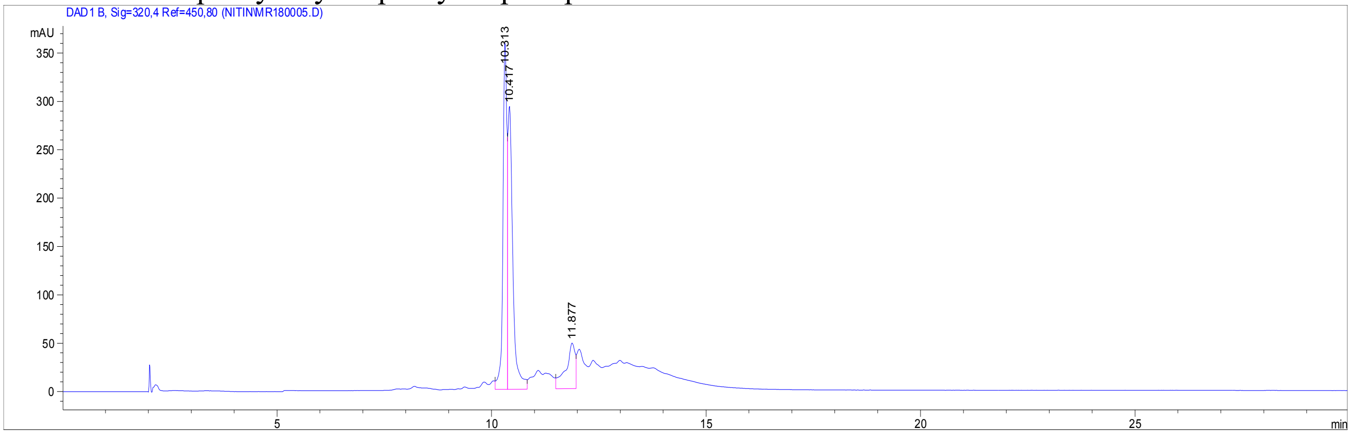

Molecule 4: Asp-Gly-Gly-Dap-Dap-Gly-Dap

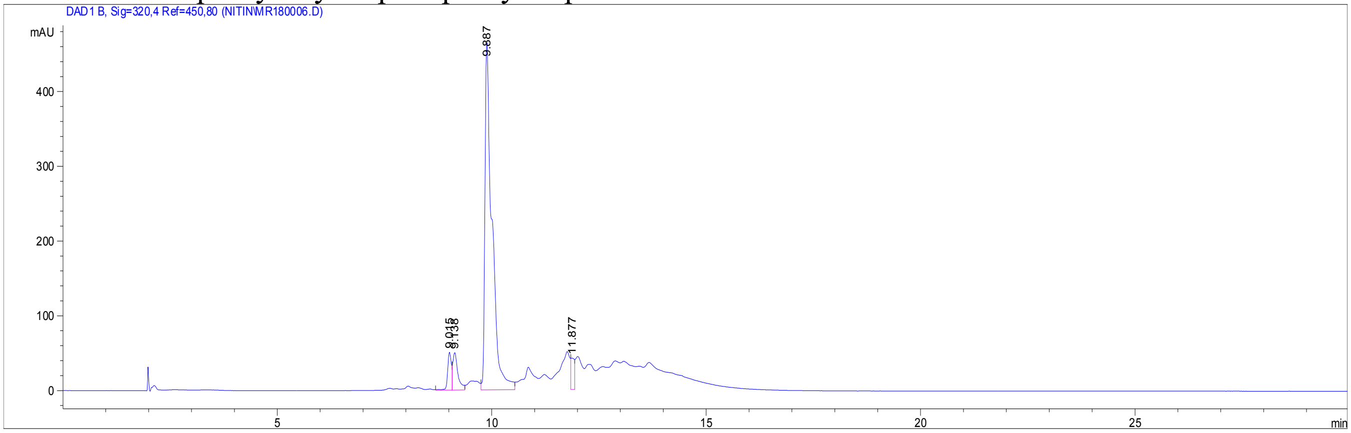

Molecule 5: Asp-Gly-Dap-Gly-Gly-Dap-Dap

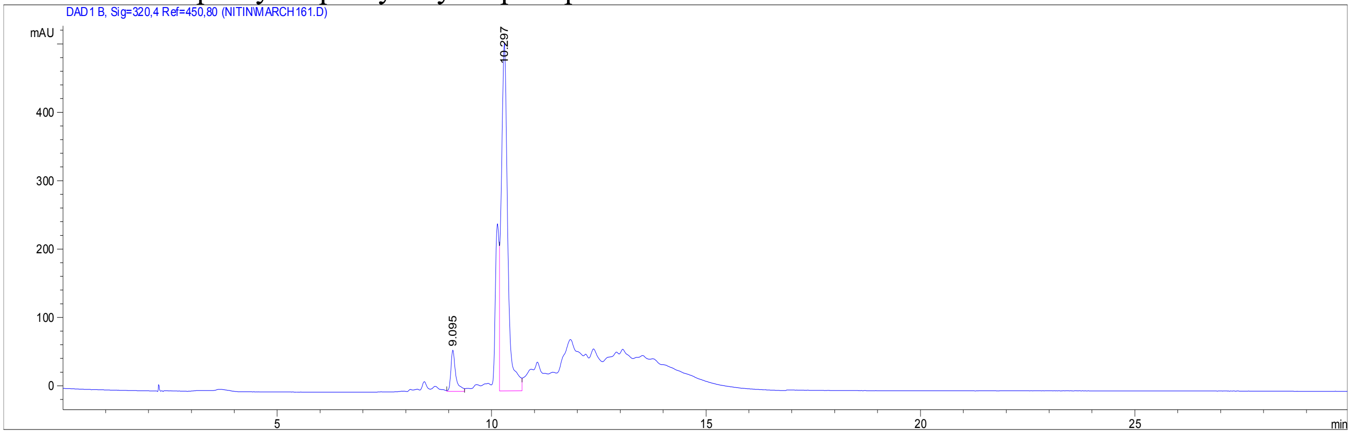

Molecule 6: Asp-Gly-Dap-Gly-Dap-Gly-Dap

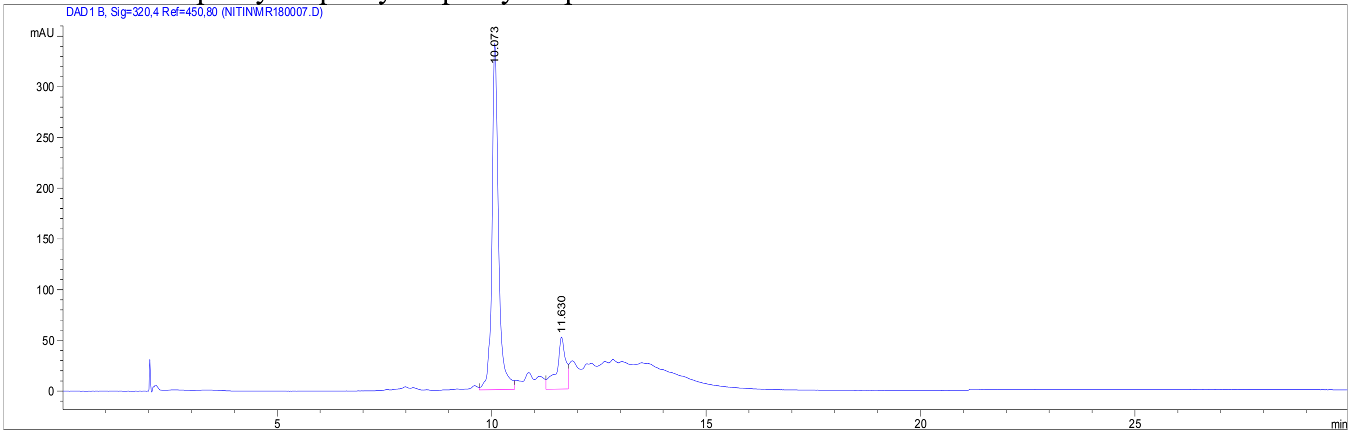

Molecule 7: Asp-Gly-Dap-Dap-Gly-Gly-Dap

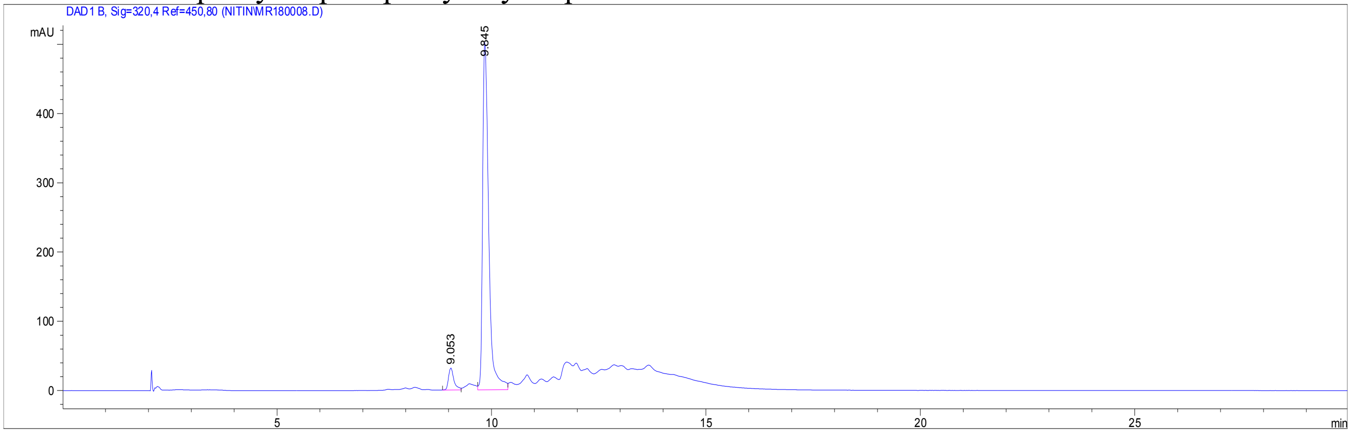

Molecule 8: Asp-Dap-Gly-Gly-Gly-Dap-Dap

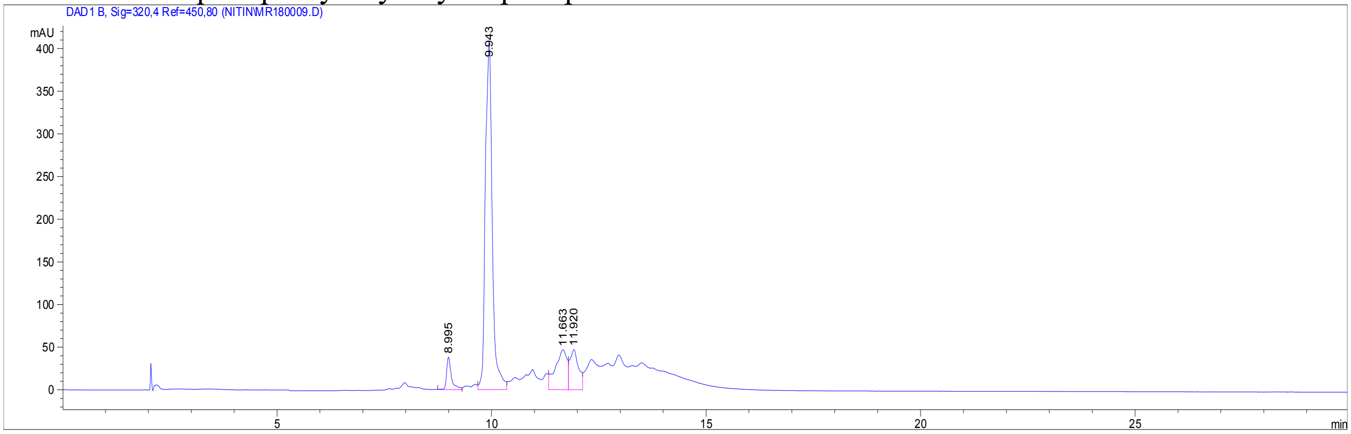

Molecule 9: Asp-Dap-Gly-Gly-Dap-Gly-Dap

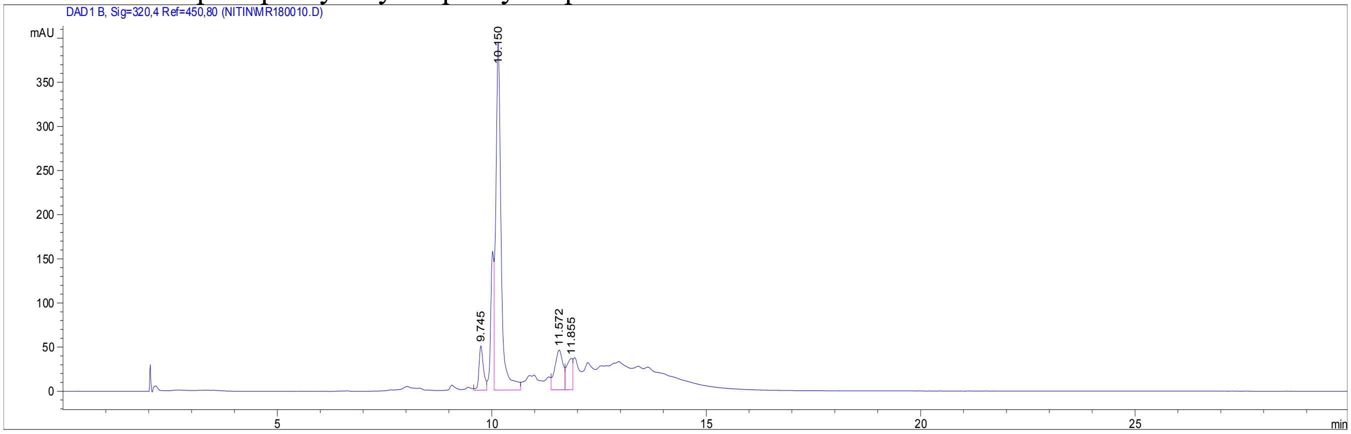

Molecule 10: Included in main paper (Representative data)

Molecule 11: Asp-Dap-Dap-Gly-Gly-Gly-Dap

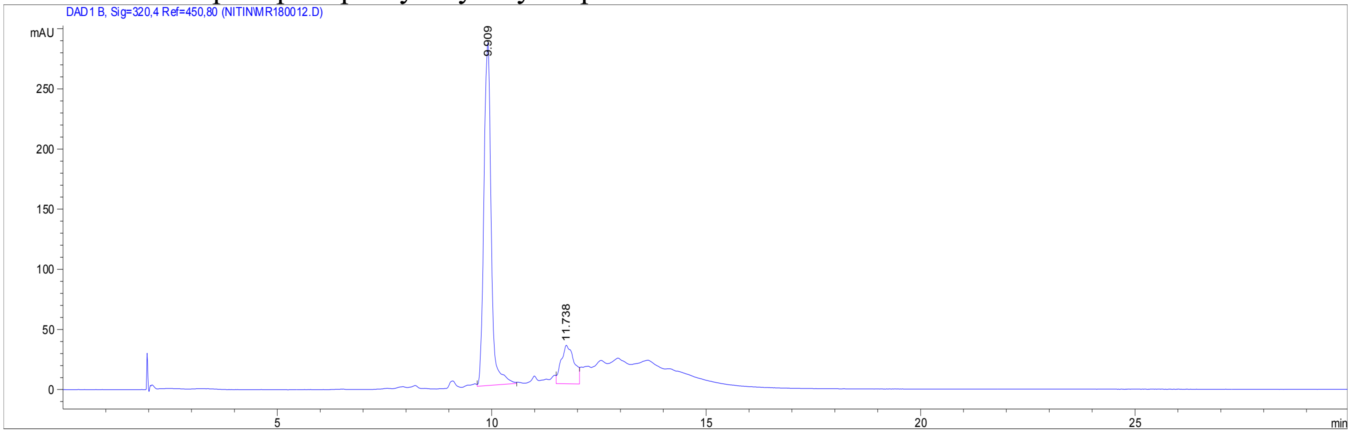

Molecule 12: Asp-Gly-Dap-Dap-Gly-Dap-Dap

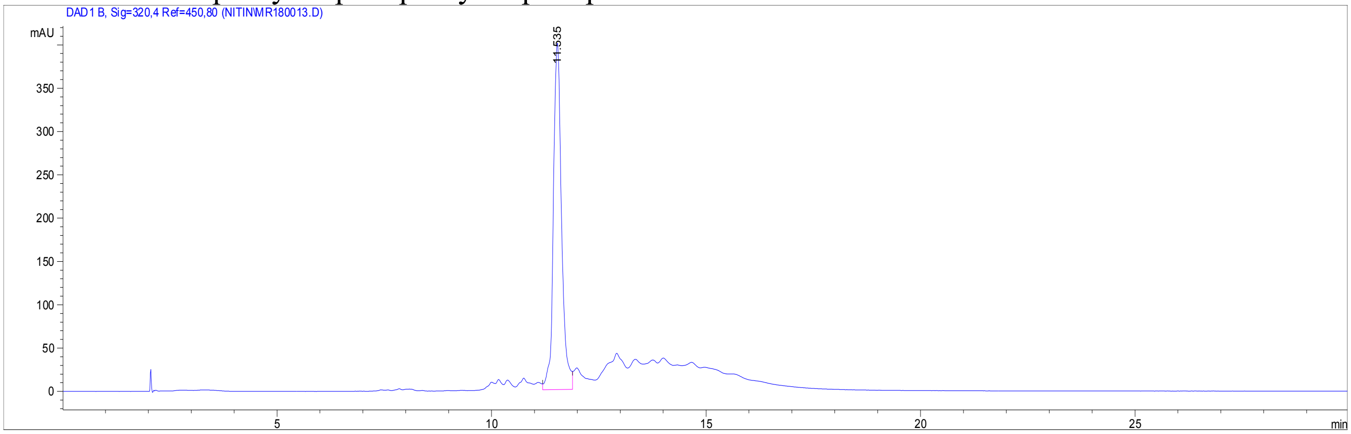

Molecule 13: Asp-Gly-Dap-Dap-Dap-Gly-Dap

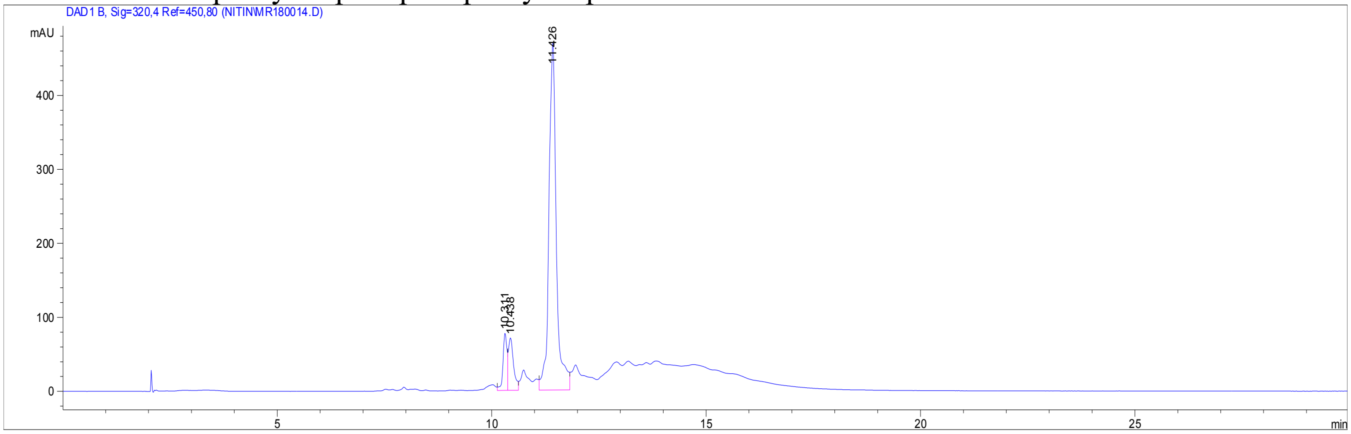

Molecule 14: Asp-Gly-Gly-Dap-Dap-Dap-Dap

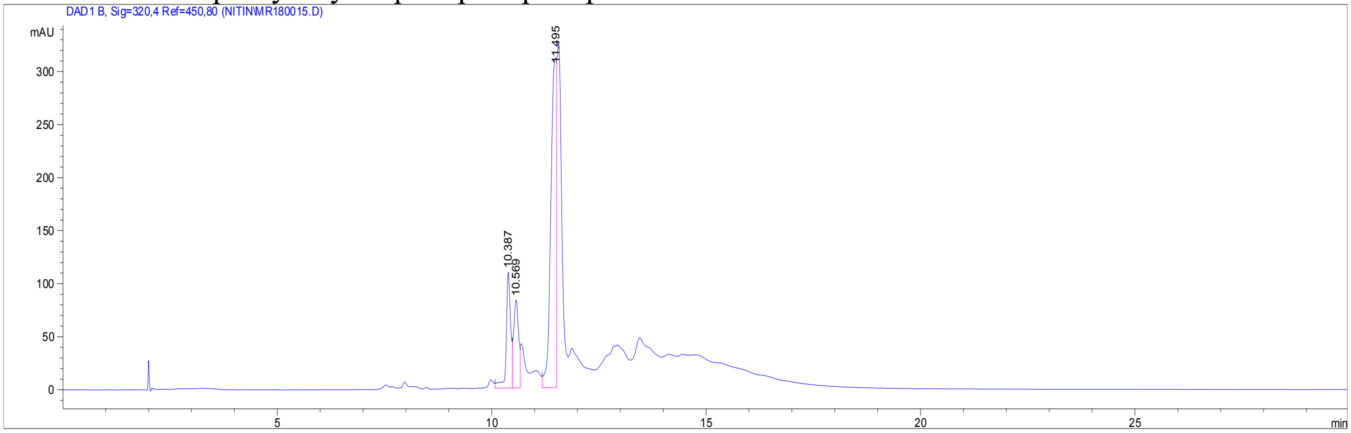

Molecule 15: Asp-Gly-Dap-Gly-Dap-Dap-Dap

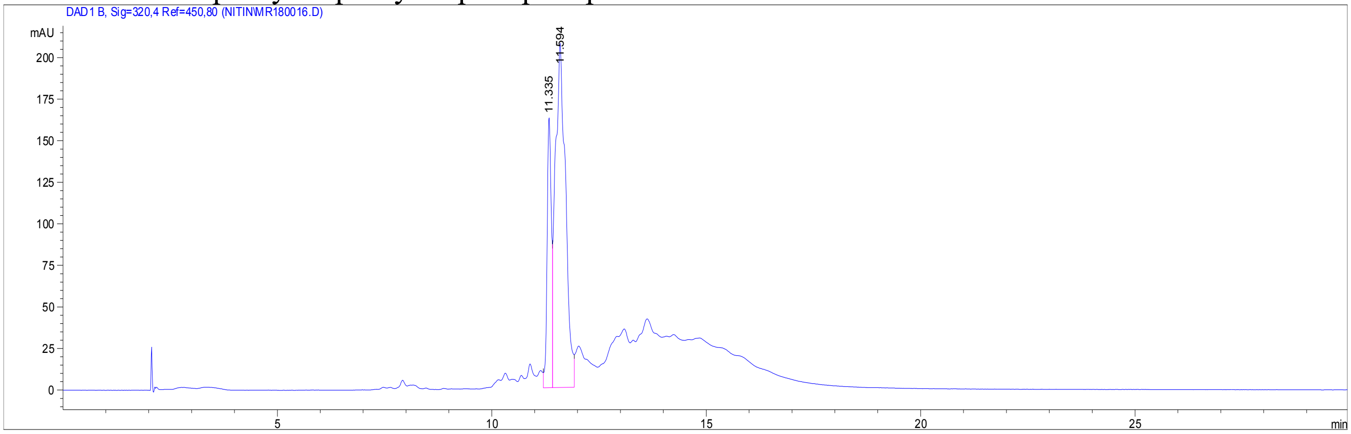

Molecule 16: Asp-Dap-Gly-Gly-Dap-Dap-Dap

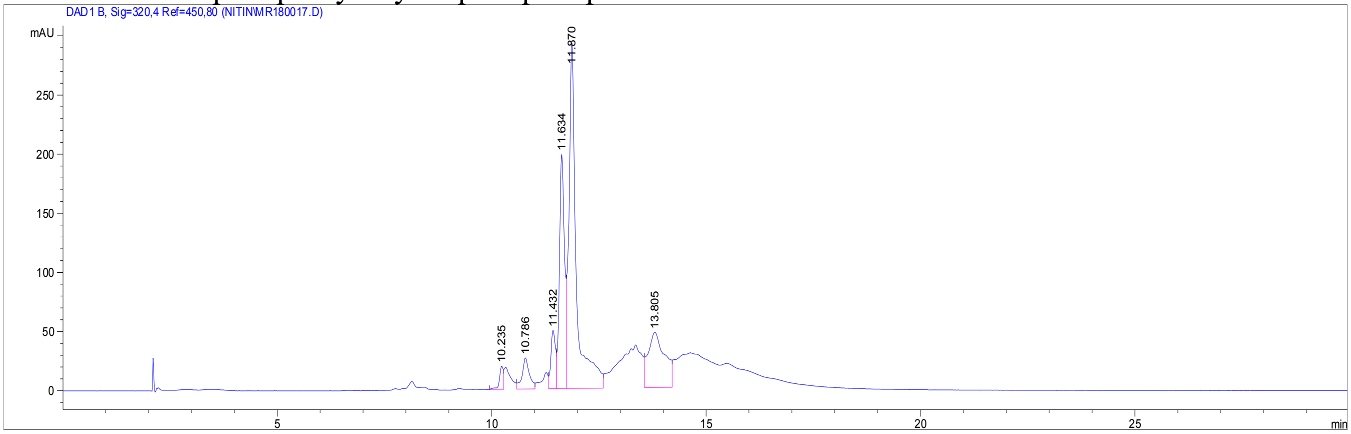

Molecule 17: Asp-Dap-Dap-Gly-Gly-Dap-Dap

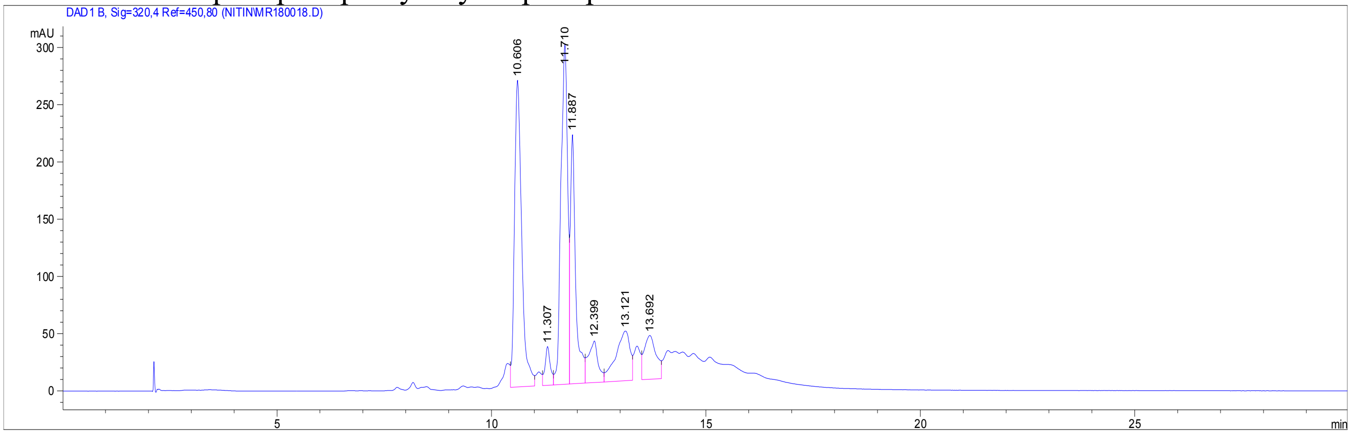

Molecule 18: Asp-Dap-Gly-Dap-Gly-Dap-Dap

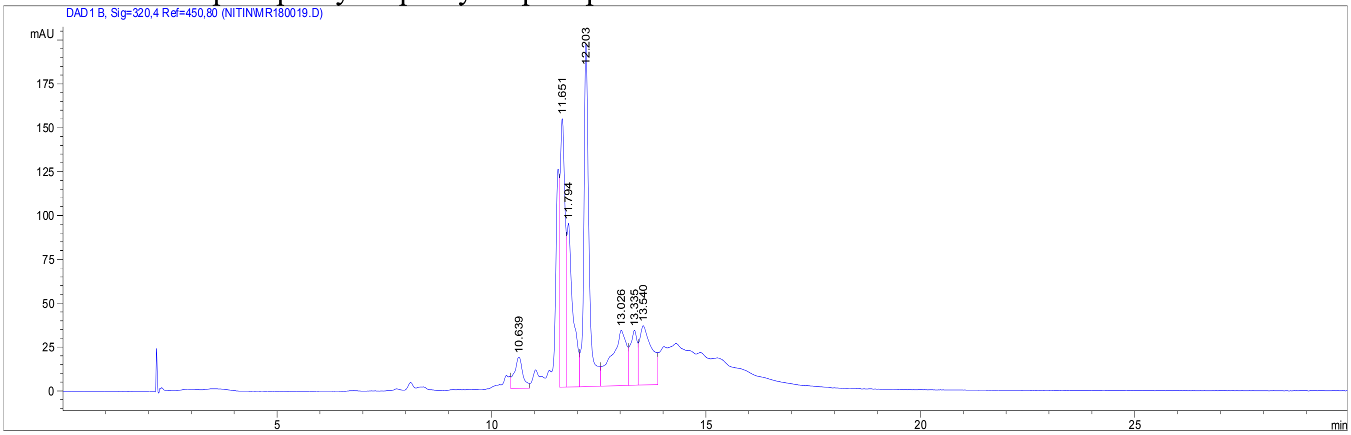

Molecule 19: Asp-Dap-Gly-Dap-Dap-Gly-Dap

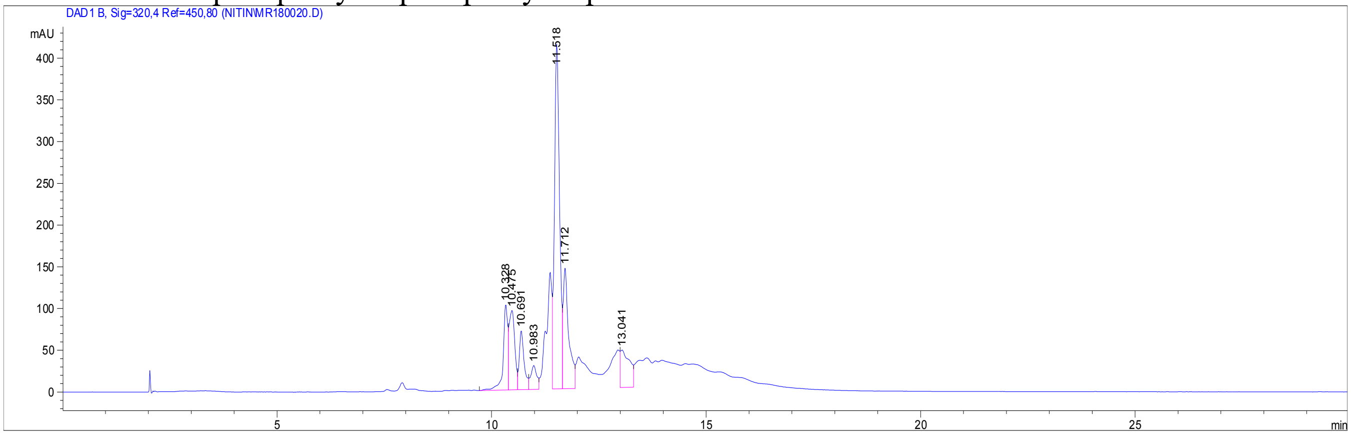

Molecule 20: Asp-Dap-Dap-Gly-Dap-Gly-Dap

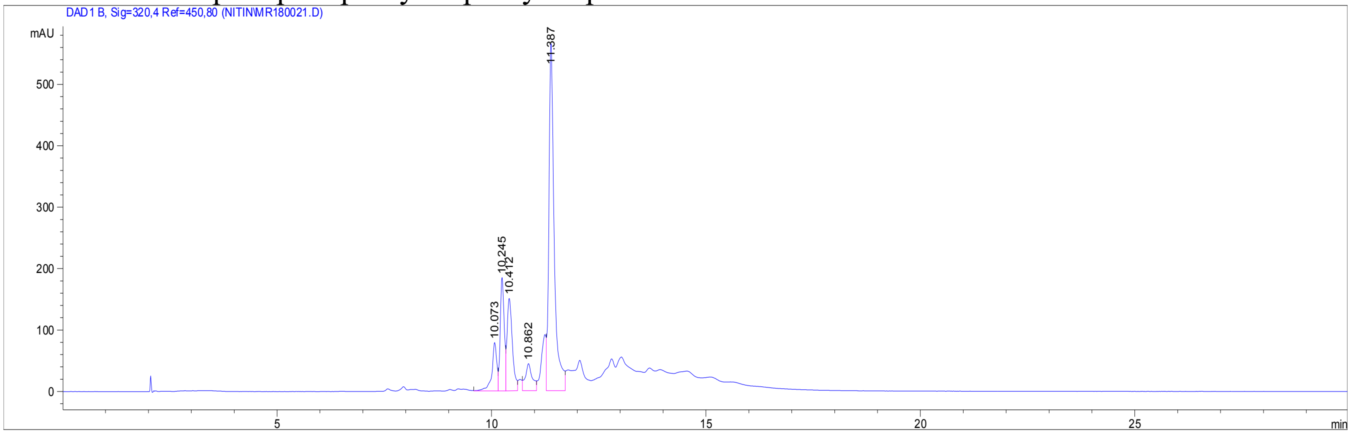

Molecule 21: Asp-Dap-Dap-Dap-Gly-Gly-Dap

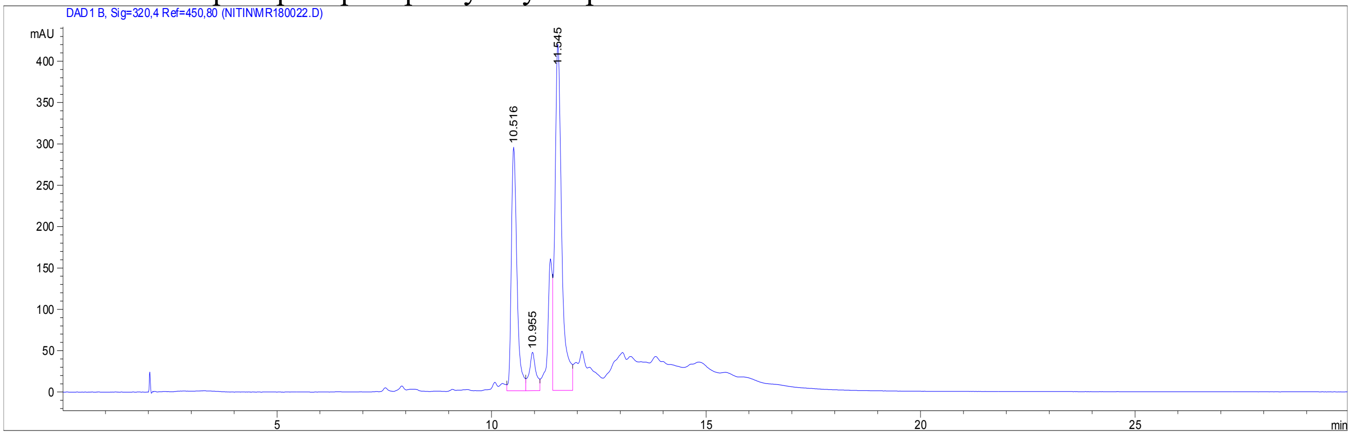

ESI-MS analysis 21 crude products obtained after TFA cleavage of the cyclic heptapeptide library.

Molecule 1

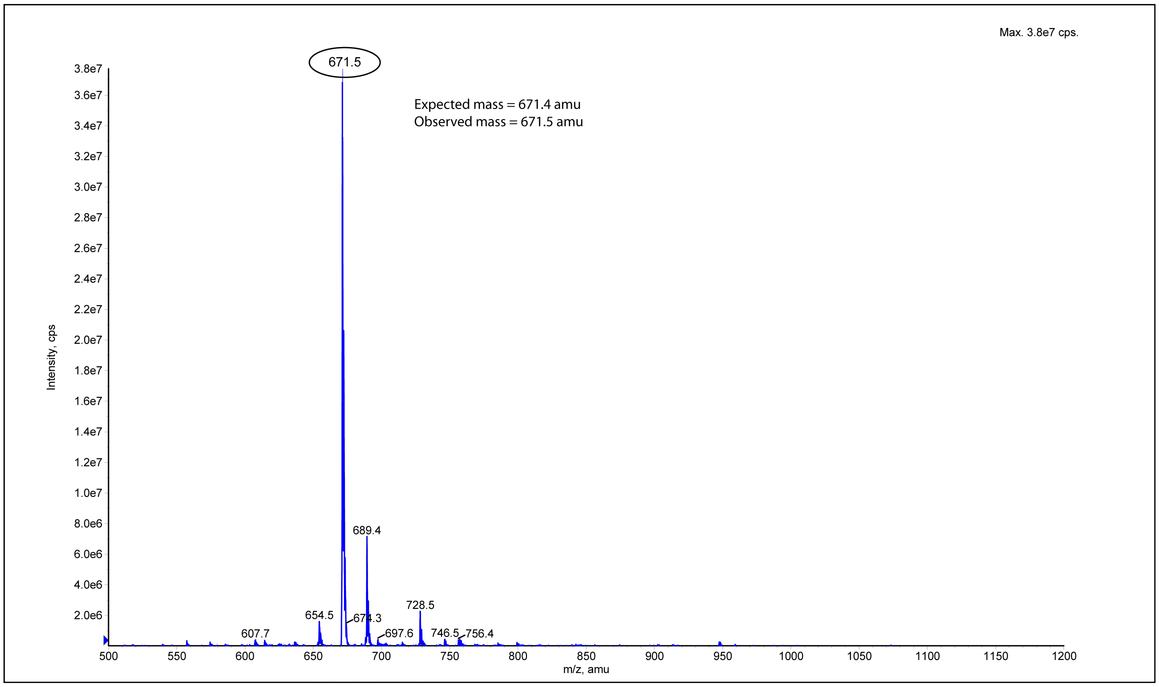

Molecule 2

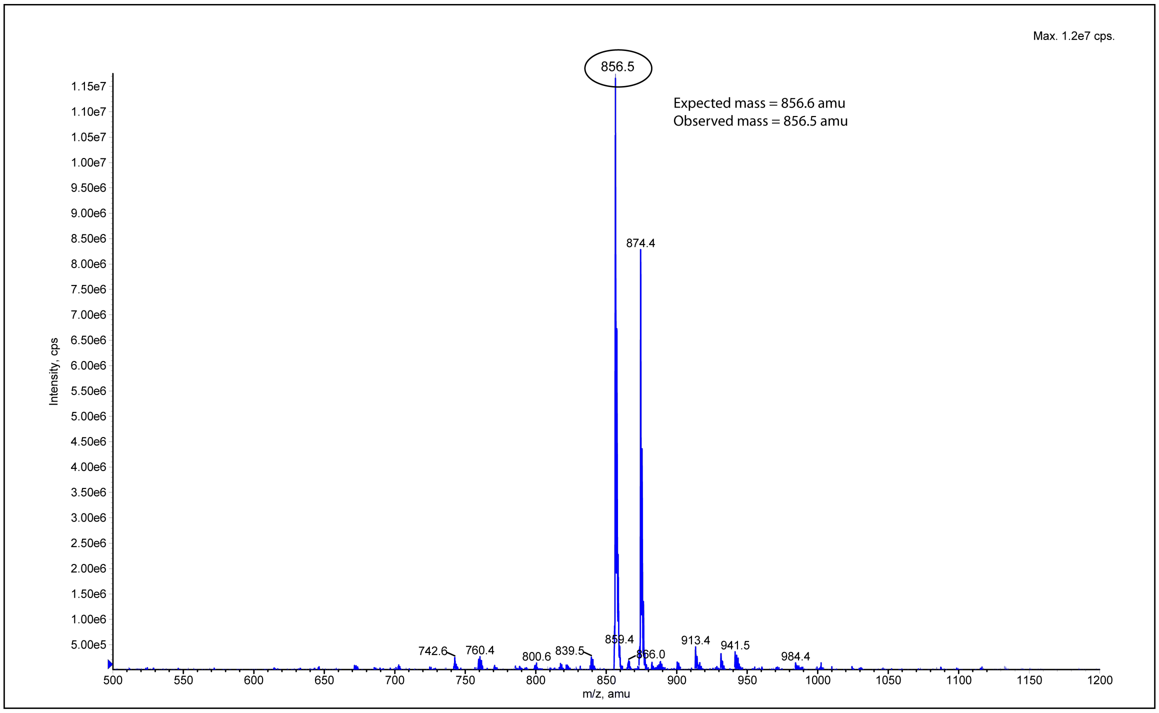

Molecule 3

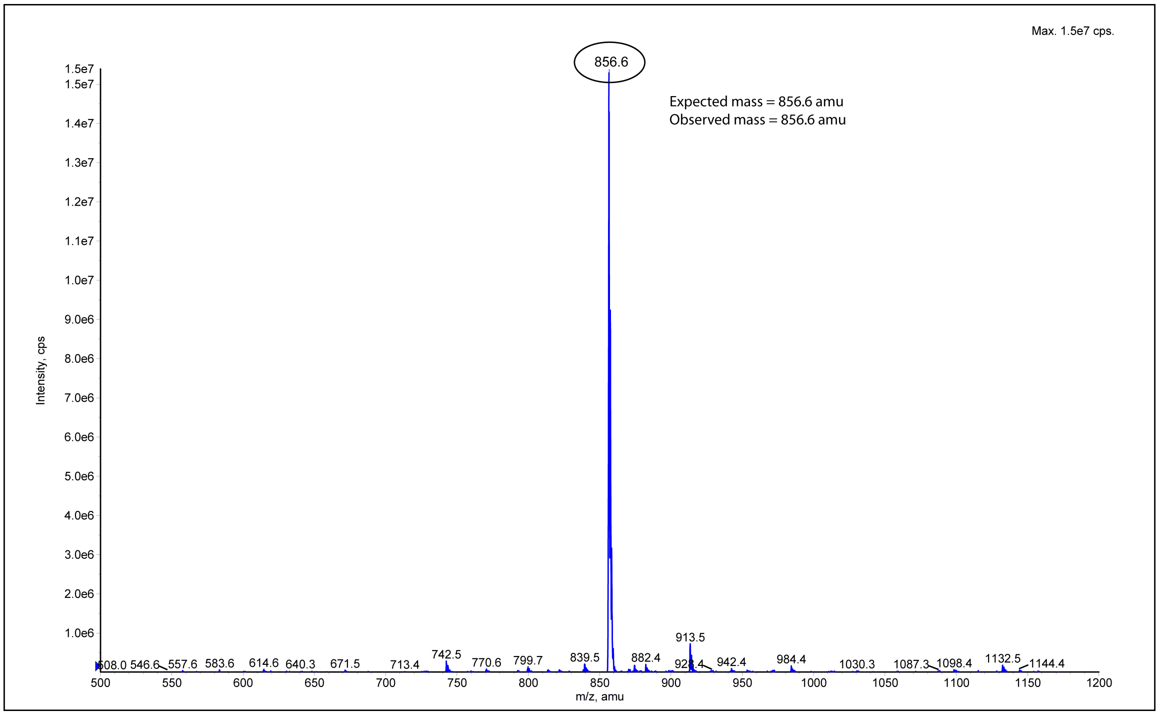

Molecule 4

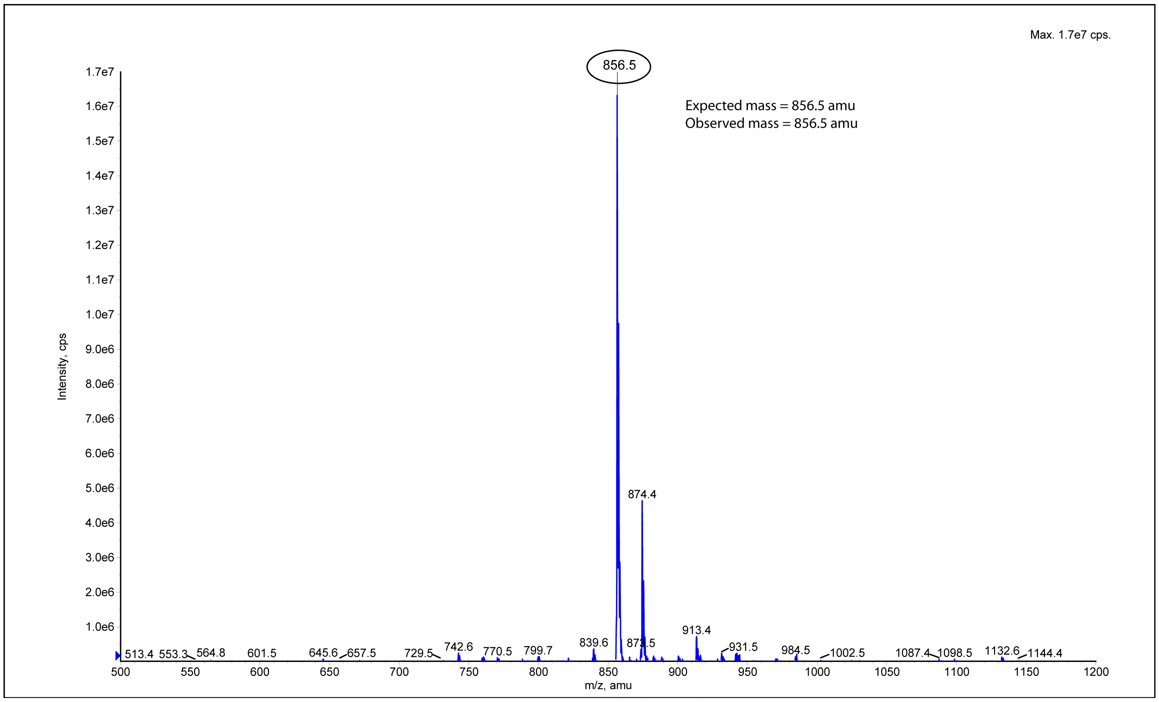

Molecule 5

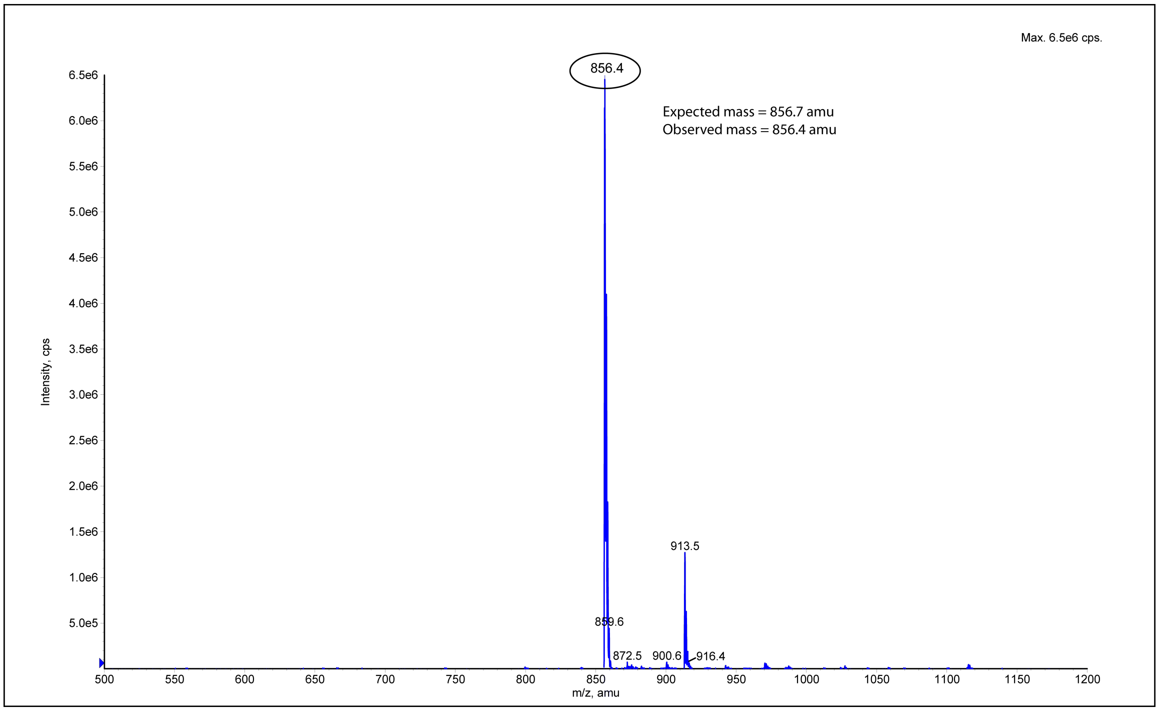

Molecule 6

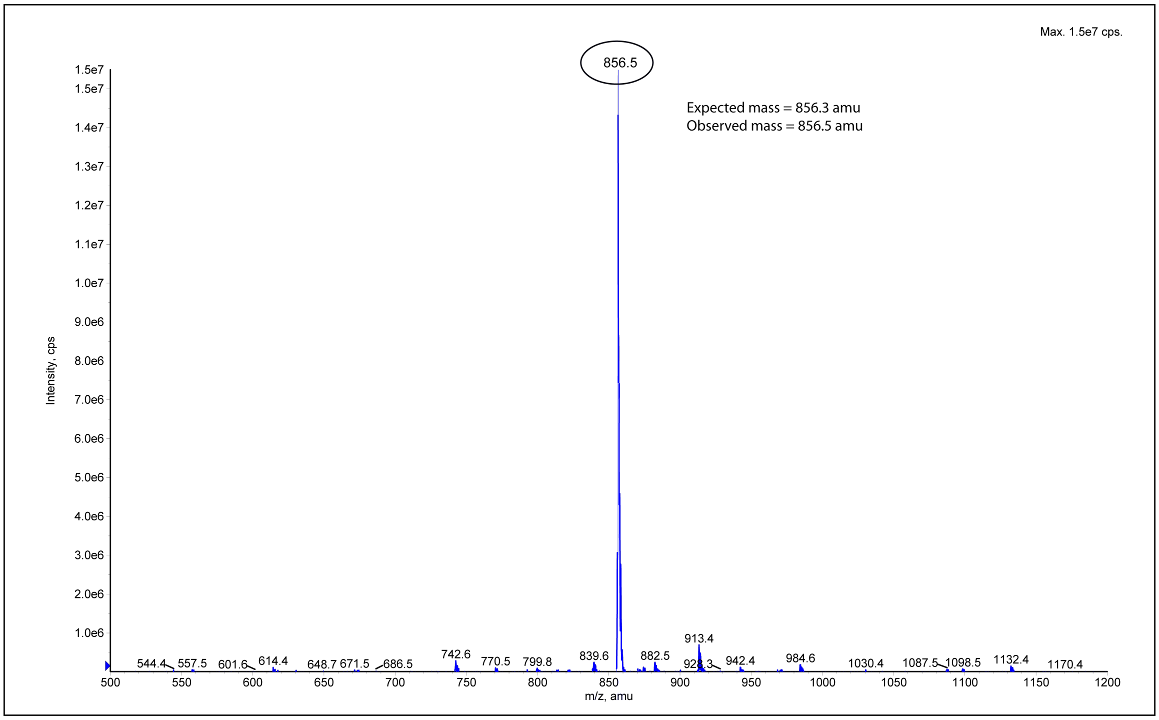

Molecule 7

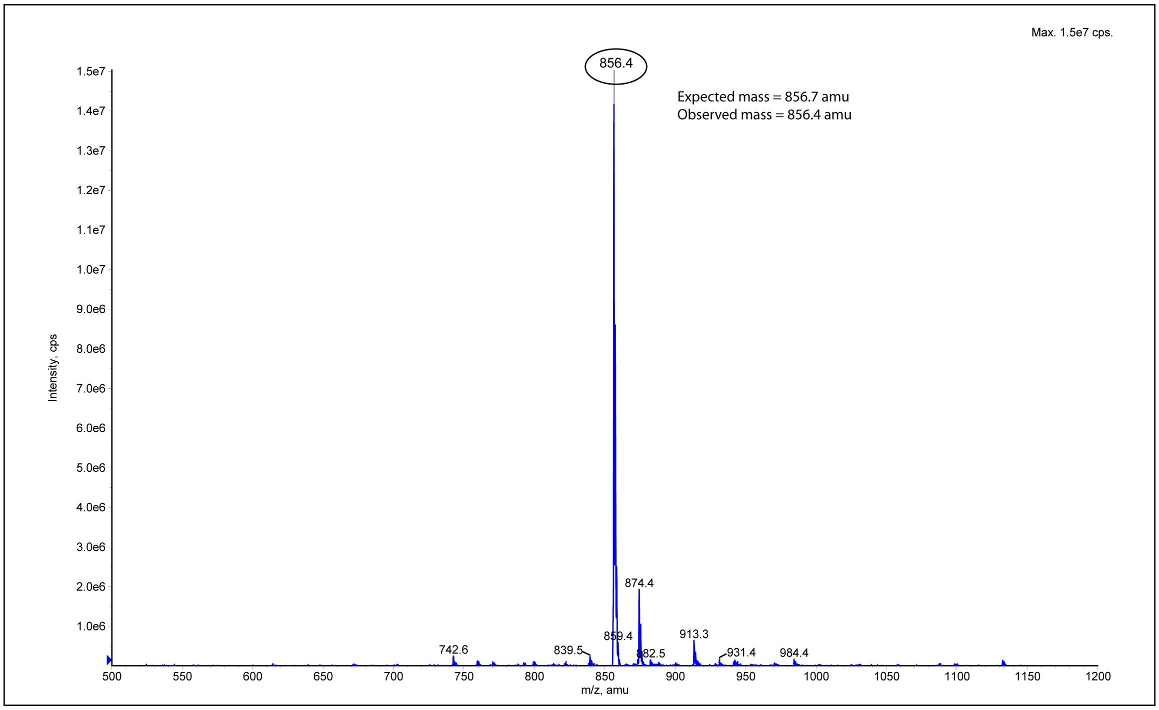

Molecule 8

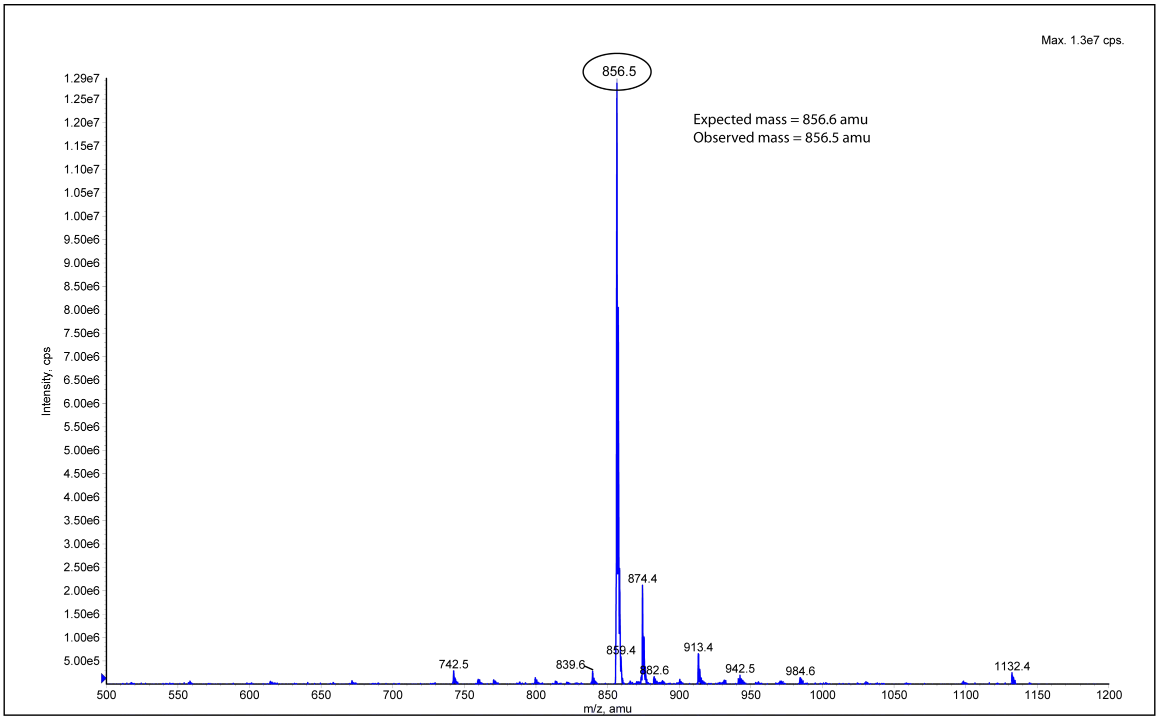

Molecule 9

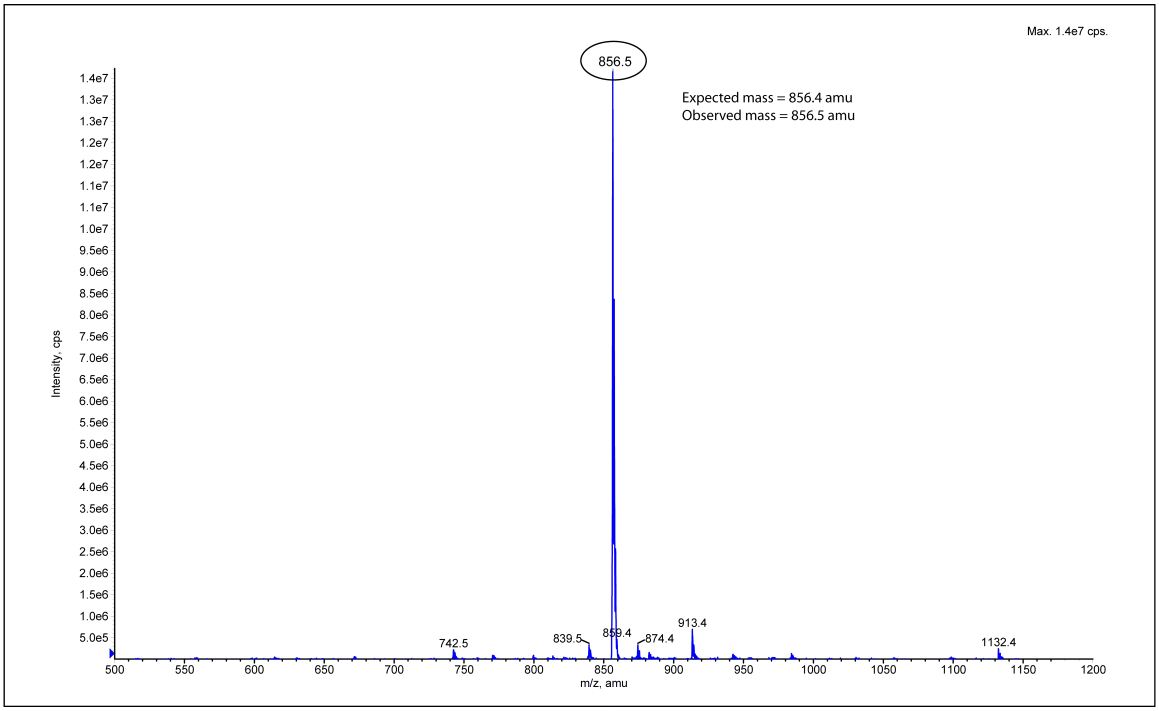

Molecule 10

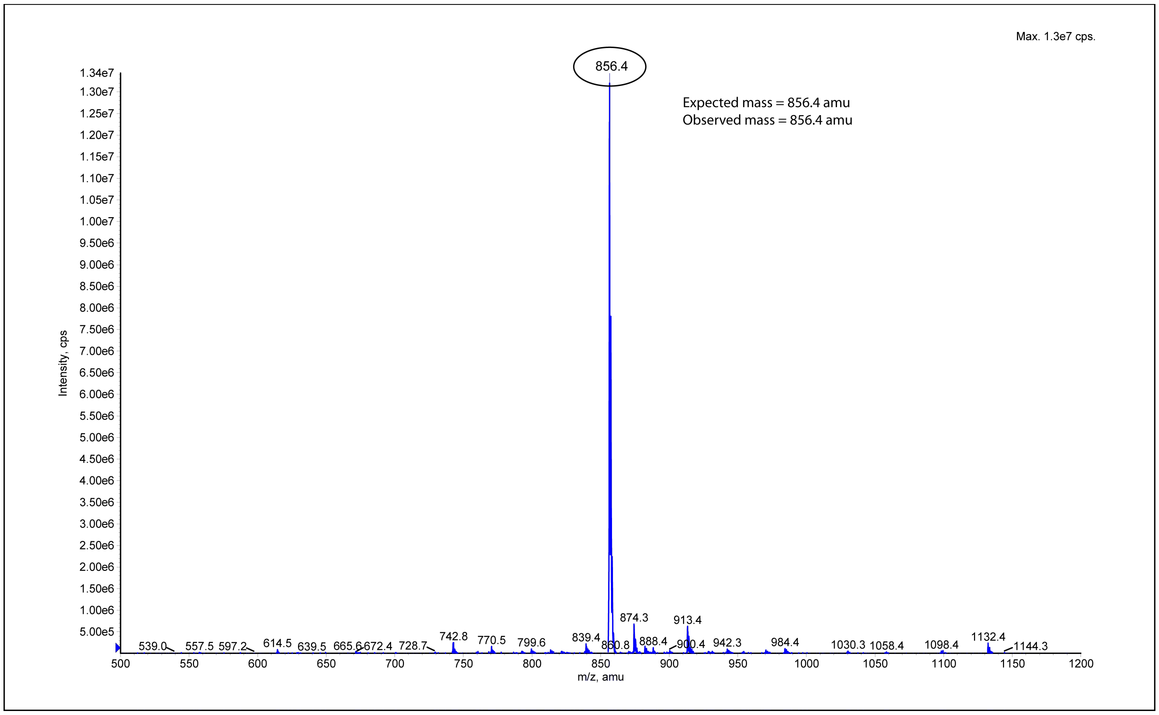

Molecule 11

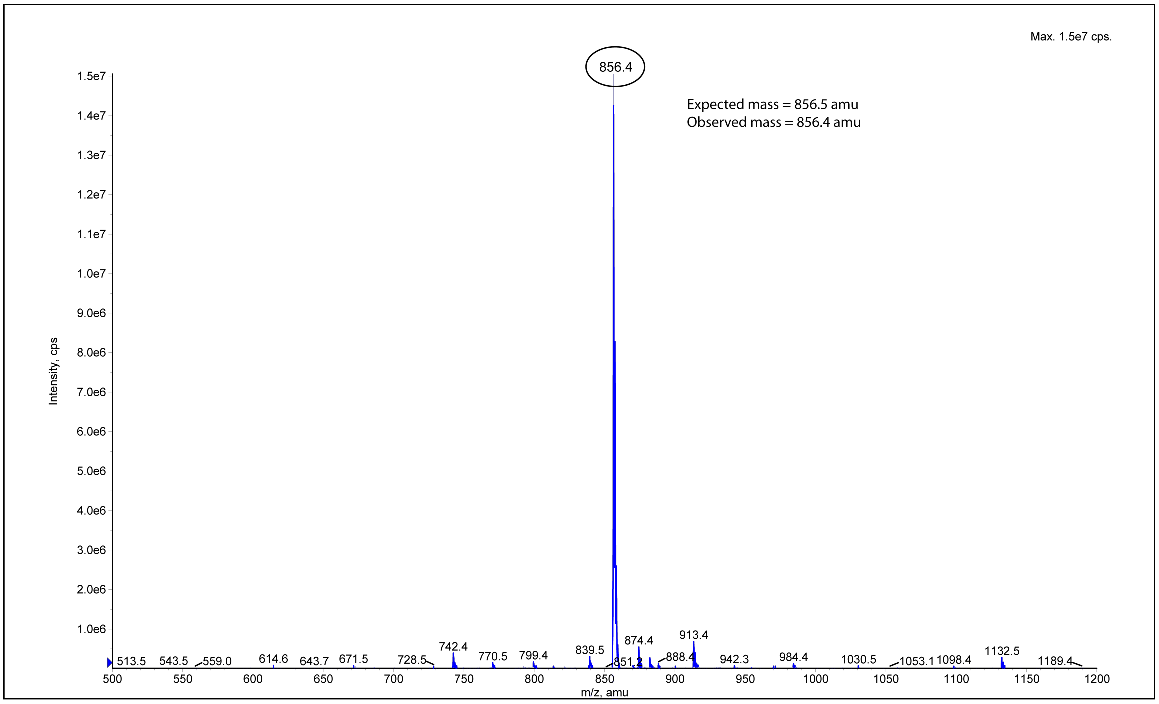

Molecule 12

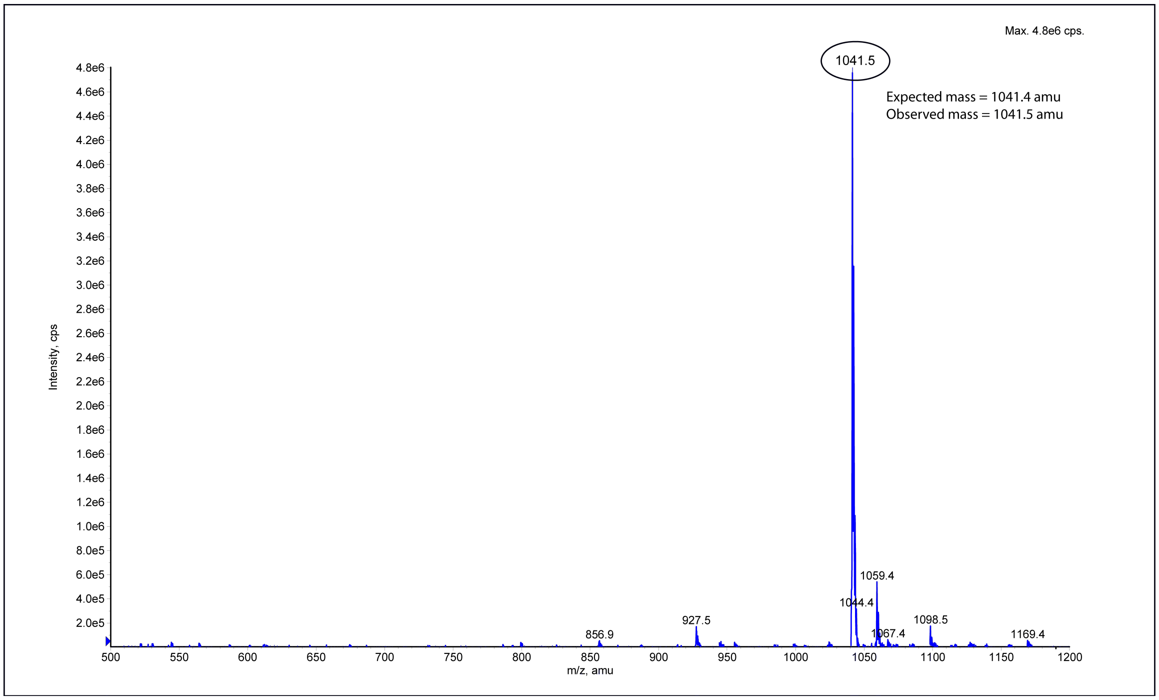

Molecule 13

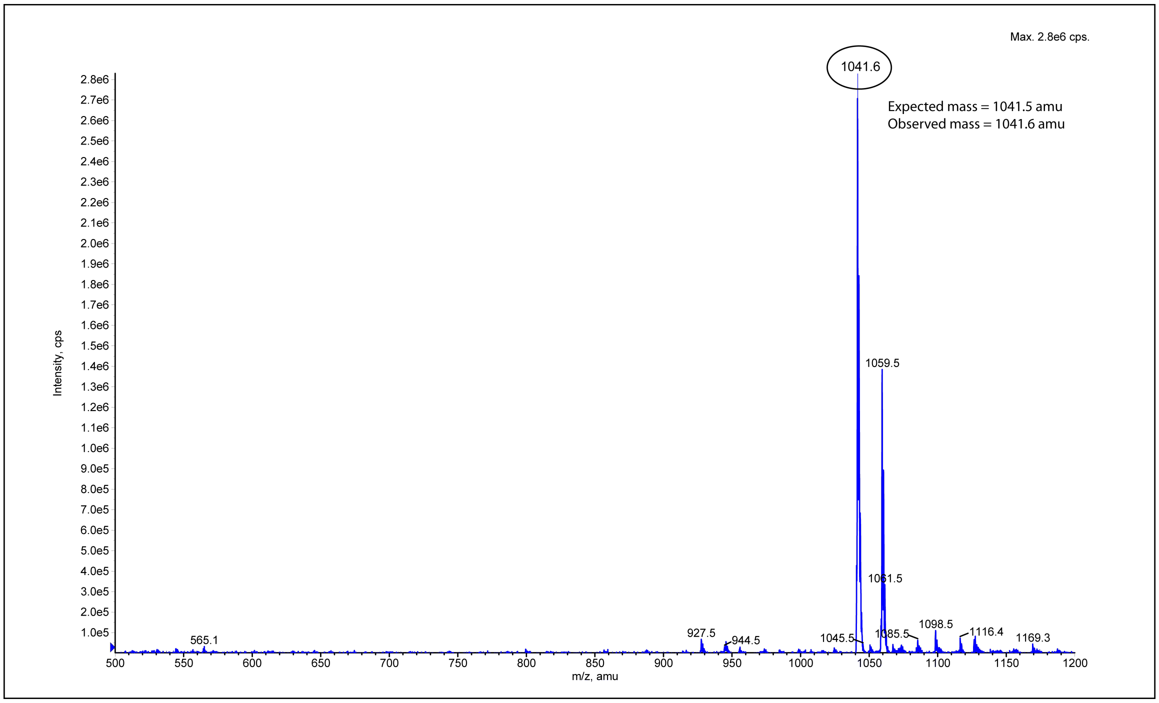

Molecule 14

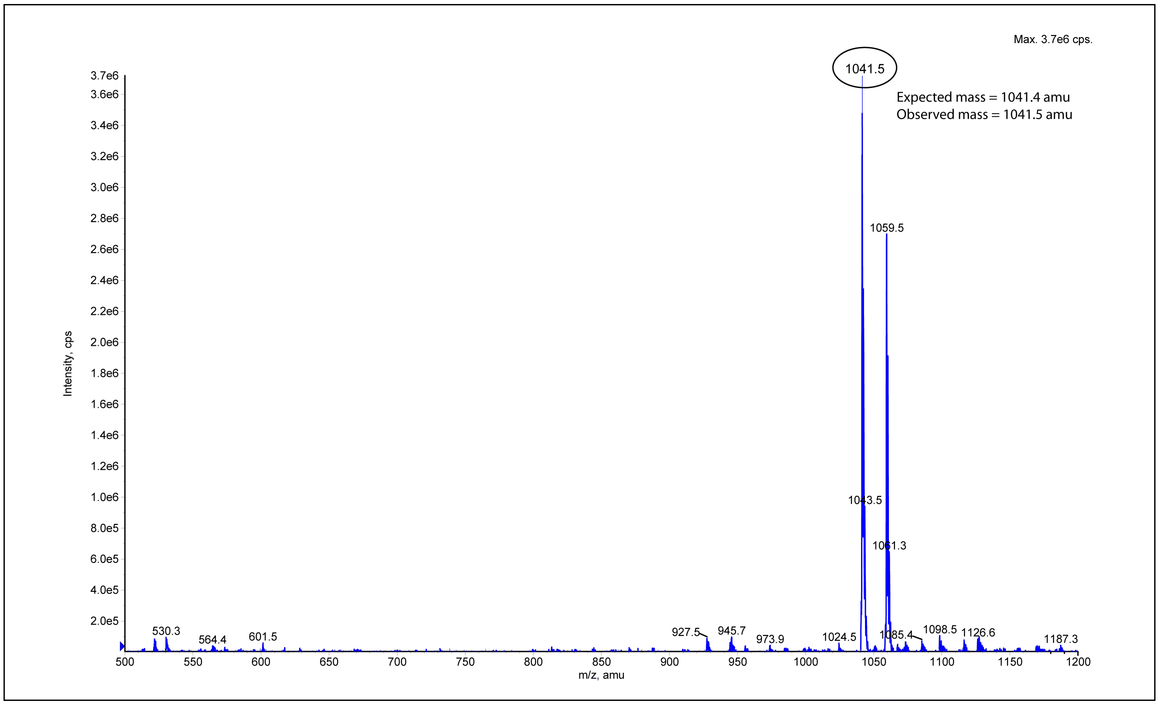

Molecule 15

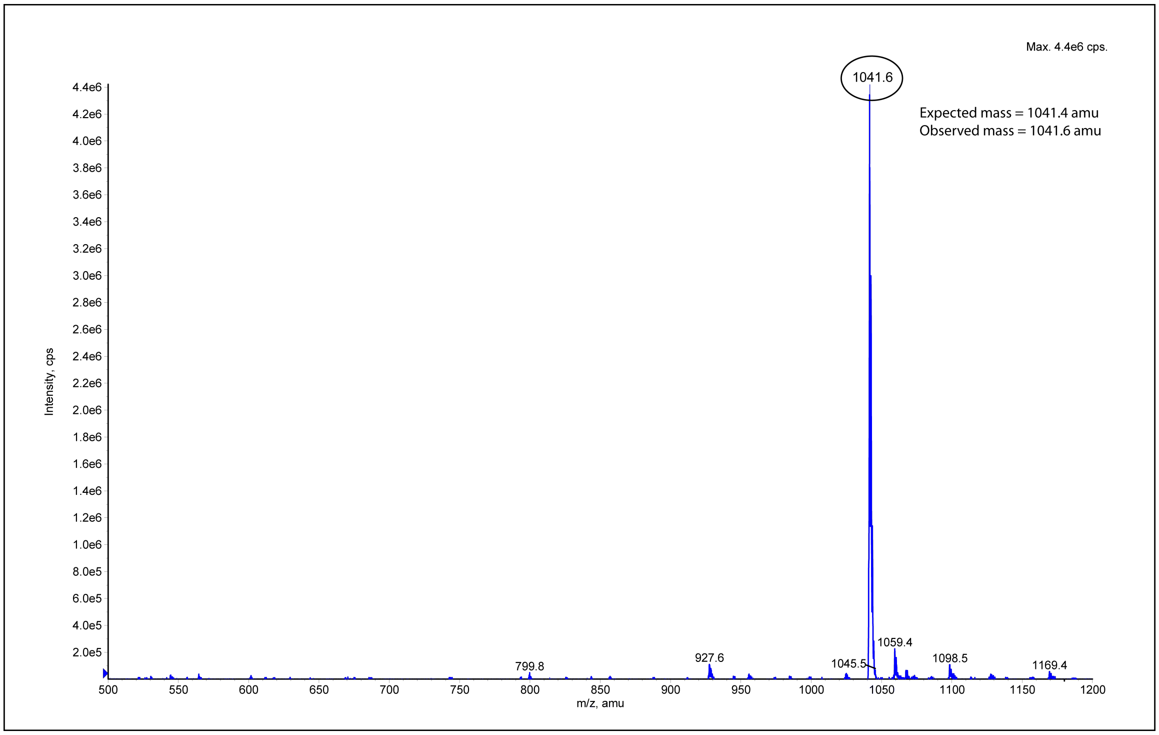

Molecule 16

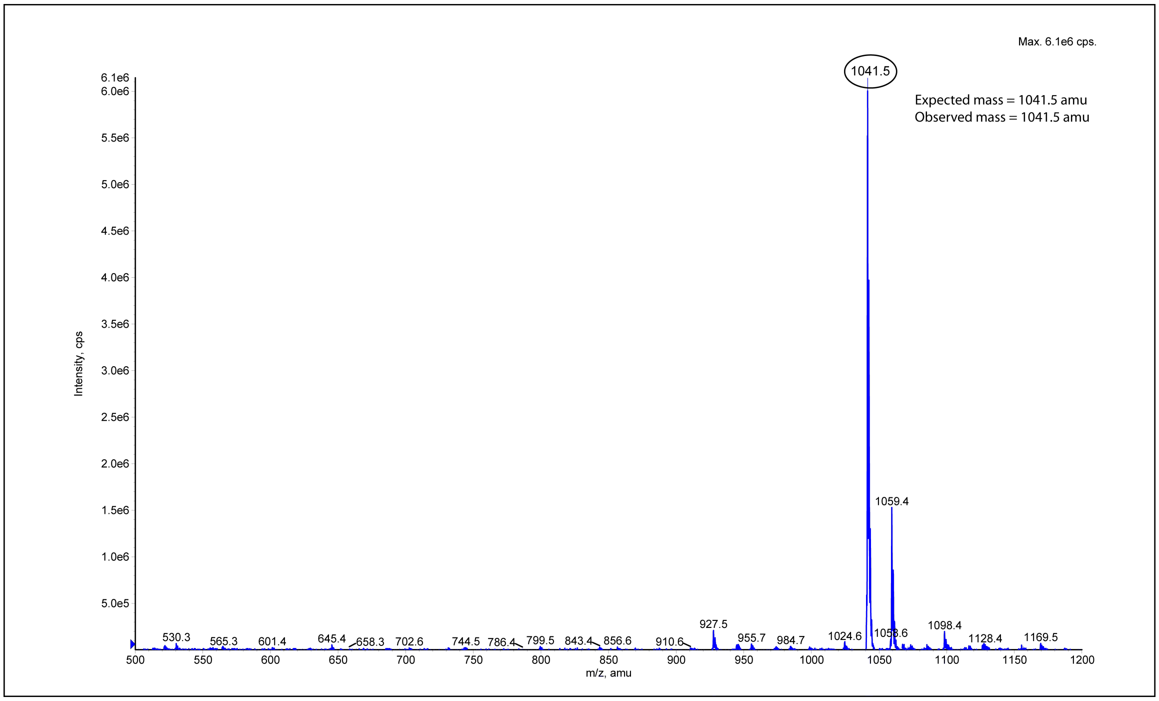

Molecule 17

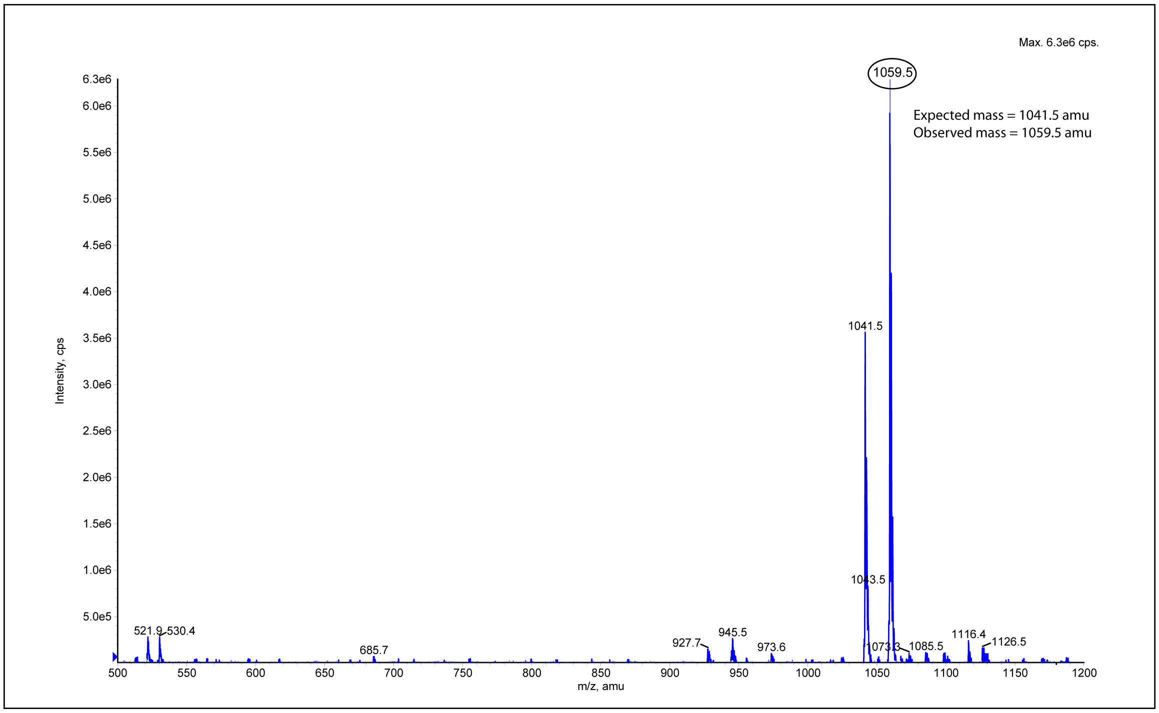

Molecule 18

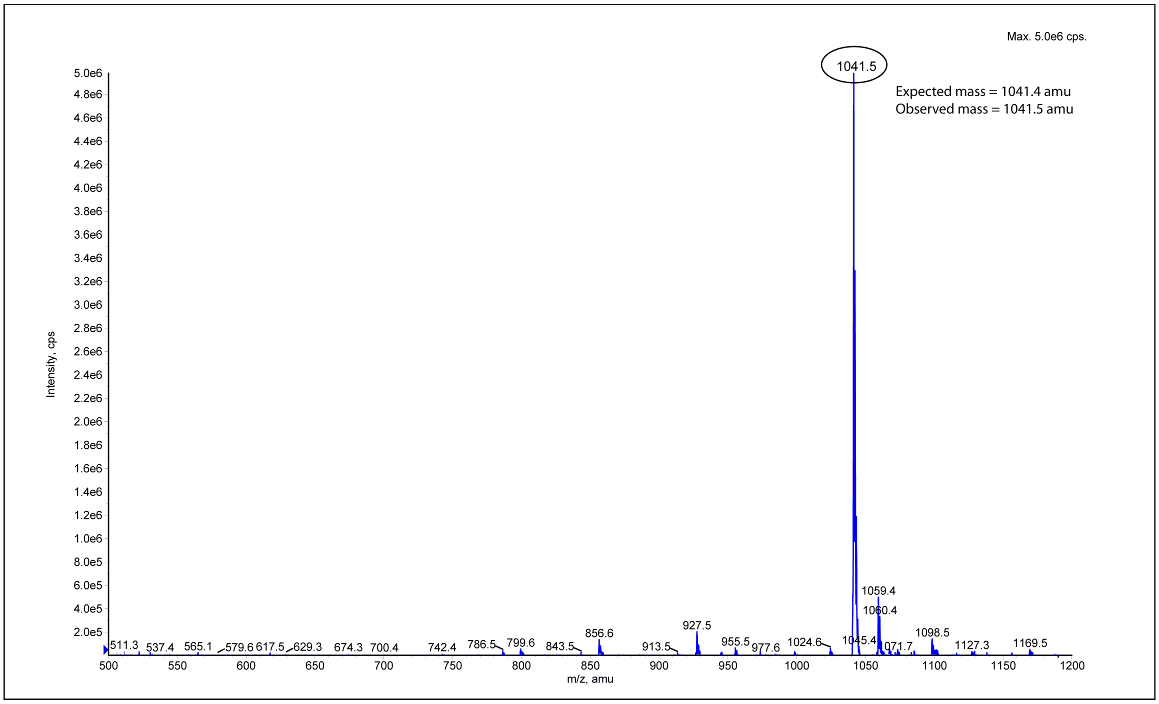

Molecule 19

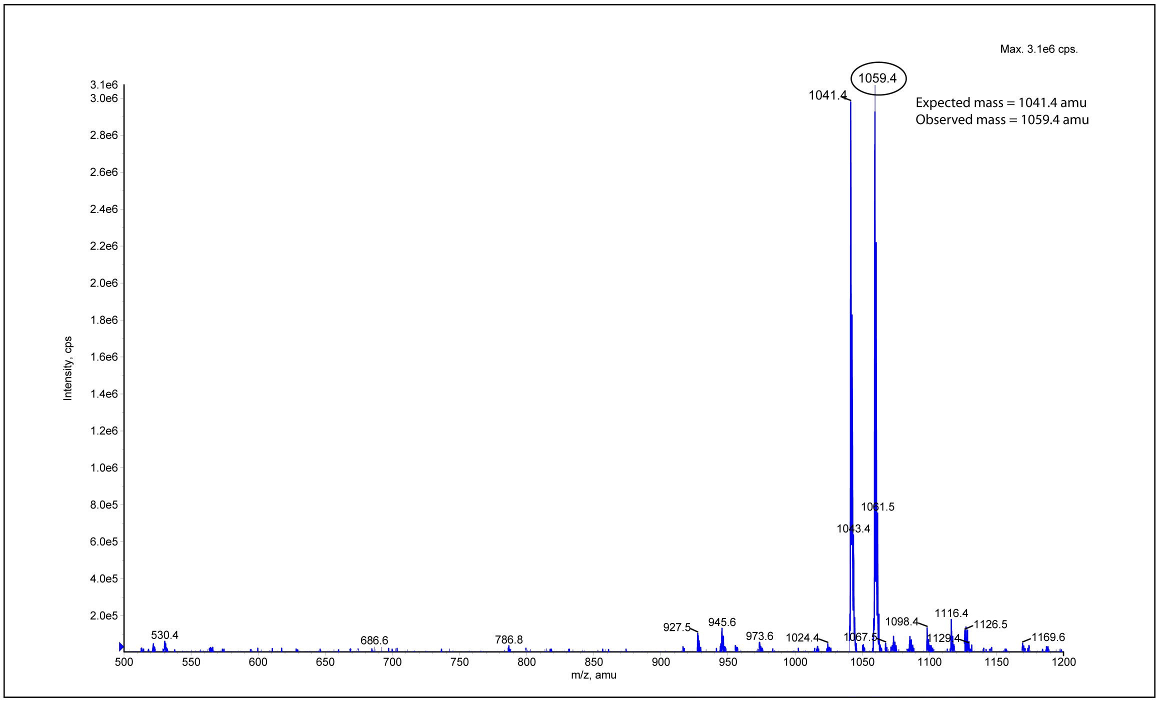

Molecule 20

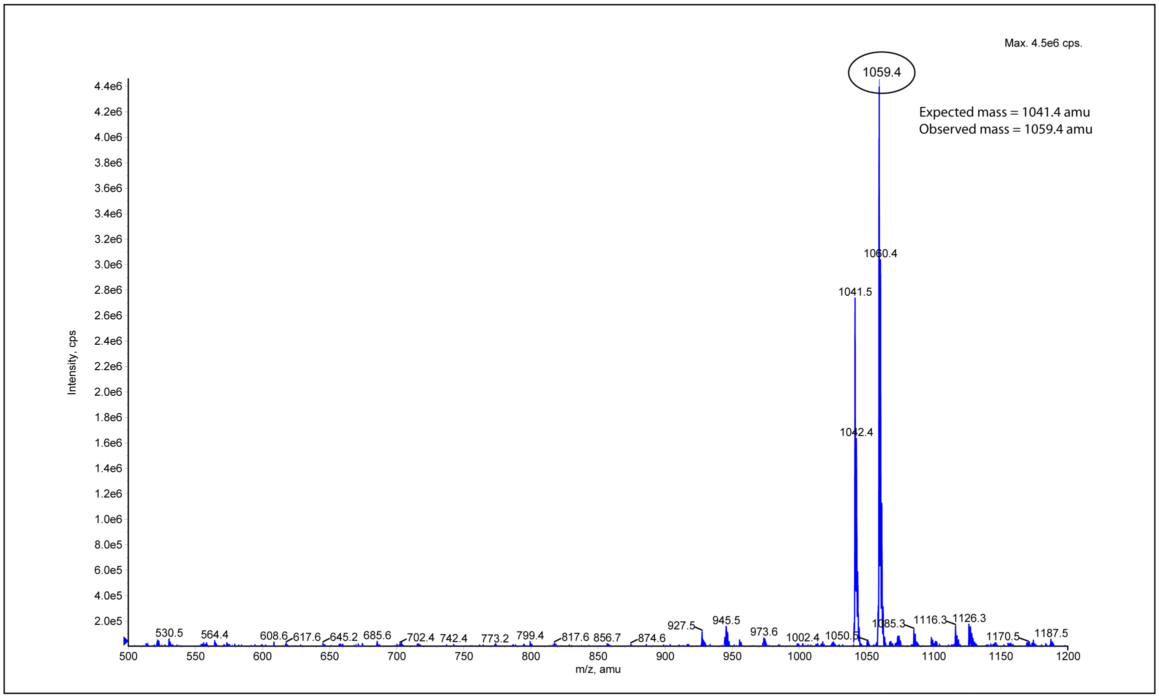

Molecule 21

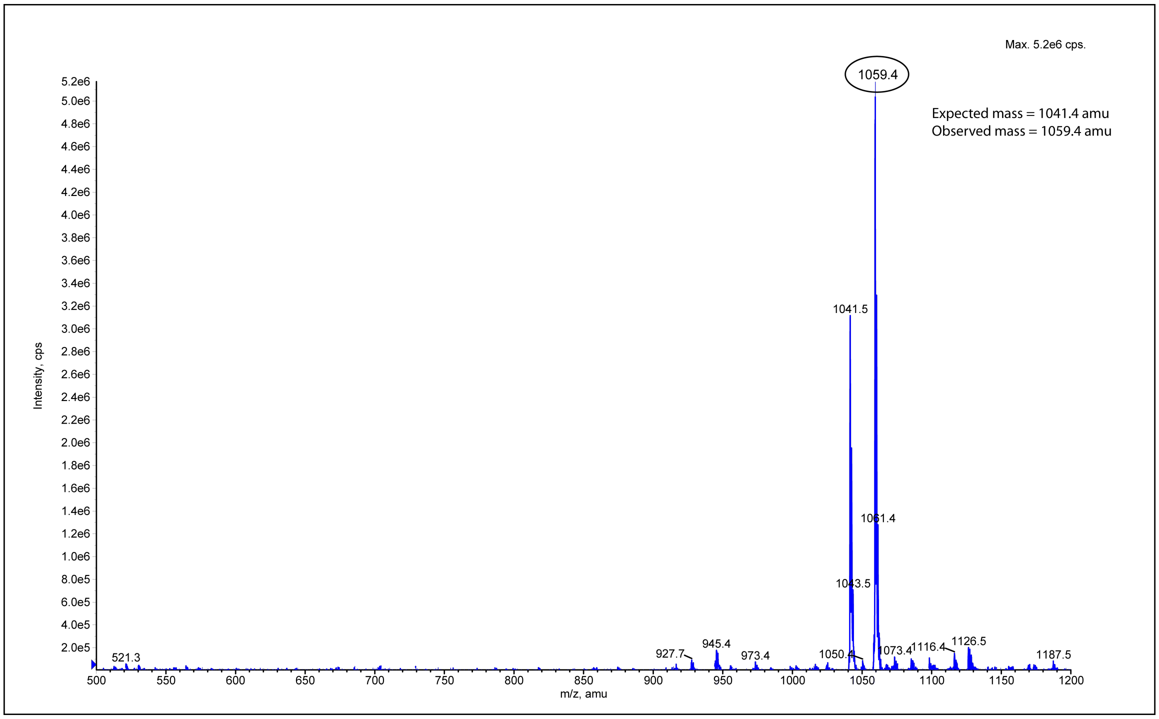

References

[1] J.J. Diaz-Mochon, L. Bialy, M. Bradley, Full orthogonality between Dde and Fmoc: The direct synthesis of PNA-peptide conjugates, Organic Letters 6(7) (2004) 1127-1129.
